# Lithium-Ion Partial Molar Entropies in Liquid, Composite, and Solid-State Electrolytes

**DOI:** 10.1021/acs.jpclett.6c00870

**Published:** 2026-06-22

**Authors:** Austin Fan, Patrick J. West, Louis Vincent Morris, Dimitrios Fraggedakis, Rachel Carter, Kelsey B. Hatzell

**Affiliations:** † Chemical and Biological Engineering, Princeton University, Princeton, New Jersey 08544, United States; ‡ Andlinger Center for Energy and the Environment, Princeton University, Princeton, New Jersey 08544, United States; ¶ Chemistry Division, 41487 Naval Research Laboratory, Washington, D.C. 20375, United States; ∥ Department of Chemical and Biological Engineering, Princeton University, Princeton, New Jersey 08544, United States; ⊥ Department of Mechanical Engineering, University of Kansas, Lawrence, Kansas 66045, United States; # Department of Mechanical and Aerospace Engineering, Princeton University, Princeton, New Jersey 08544, United States

## Abstract

Electrolyte composition, concentration, and structure can affect reaction kinetics, ion transport, and heat generation during operation. In liquid electrolyte systems, the properties of ion–solvent and ion–ion interactions strongly affect thermodynamics and electrochemistry. The change in total entropy of the electrolyte when one mole of an ion is added to an electrolyte at a constant temperature and pressure is known as the partial molar entropy of ion solvation and describes broadly how the solvation environment changes with the addition or removal of an ion. Herein, we measure the partial molar entropies of lithium-ion solvation in solid-state systems using potentiometric temperature coefficient measurements. For solid-state systems, we measure positive partial molar entropies of lithium-ion solvation of 43.3 J mol^–1^ K^–1^ for Li_6_PS_5_Cl and 26.4 J mol^–1^ K^–1^ for Li_6.5_La_3_Zr_1.5_T_0.5_O_12_. In contrast, the liquid electrolyte 1 M LiPF_6_ in 1:1 vol % EC:DEC exhibits a negative partial molar entropy of −76.0 J mol^–1^ K^–1^. A one-dimensional analytical model is developed to elucidate how the temperature coefficient is influenced by the multiple interfaces formed when an inorganic single-ion conductor is combined with a binary liquid electrolyte, providing a unified framework for understanding the partial molar entropies of ion solvation across a wide range of electrochemical systems.

## Graphical abstract

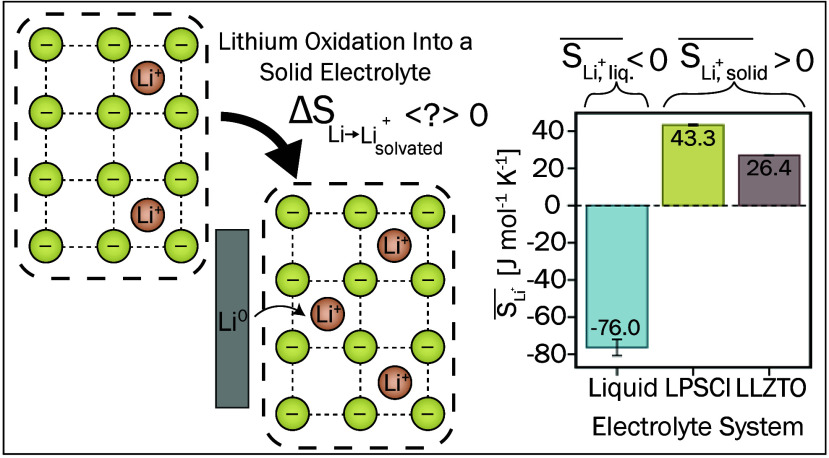

Within a battery, the electrolyte serves a crucial role as a simultaneous electron-insulating and ion-conducting layer. During traditional lithium-ion battery operation, liquid electrolytes solvate lithium ions oxidized from one electrode, transport the lithium ion to the other electrode, and desolvate the lithium ion for subsequent reduction. Given the interplay between (de)­solvation and the charge-transfer processes occurring at the electrode/electrolyte interface, understanding the thermodynamics of the (de)­solvation process, which is composed of the enthalpy and entropy of solvation, is necessary to characterize electrolyte and battery performance.^1^ For example, in batteries with liquid electrolytes, the solvation process can affect electrolyte electrochemical stability,^2,3^ thermal stability,^4,5^ and formed solid-electrolyte interphase composition and properties.^6^ Furthermore, the entropy of (de)­solvation is a key parameter in reversible heat generation during battery operation,^7^ providing insightful data for designing their thermal management systems.^8^ Recently, a substantial amount of focus has been placed on characterizing the entropy of lithium-ion solvation. Although enthalpy accounts for the majority of the free energy of (de)­solvation, the entropy of (de)­solvation has received increased interest for its role in linking temperature and corresponding cell effects as well as providing insight into the solvation structures and screening properties of the salts and solvents.^1,9^

Solid-state electrolytes have received growing interest for their ability to enable energy-dense lithium–metal battery electrodes and improve operational safety.^10–12^ Considering the impacts of the (de)­solvation process on liquid-electrolyte battery performance, understanding the thermodynamics, and, in particular, the entropy of the analogous “(de)­solvation” process in these solid-state electrolytes, is of interest. In liquid electrolytes, the thermodynamic properties of lithium-ion solvation are typically calculated using first-principles methods.^13–15^ However, similar first-principles calculations that were used to find the energies of lithium-ion solvation in liquid systems have been sparsely applied to solid-state electrolytes. Rather, calorimetric and various first-principles calculation methods in the solid-state focus on characterizing system-wide free energy values.^16,17^ While these calculations are important for understanding stability and ionic conductivity,^18^ they do not explicitly address the thermodynamics of lithium-ion (de)­solvation in these electrolytes. Electro-analytical methods, such as potentiometric methods, are therefore essential for achieving a more complete understanding of this process.

Potentiometric entropy experiments measure the entropies of electrochemical processes occurring in electrodes and electrolytes. In these experiments, the open-circuit voltage of an electrochemical cell is measured as the applied temperature conditions are varied.^19,20^ Many studies have focused on isothermal potentiometric entropy measurements, whereby the change in the Gibbs Free Energy of the cell, Δ*G*_cell_, with respect to temperature is related to the full-cell entropy of reaction, Δ*S*_rxn_, by^9,21^

$$\Delta S_{\mathrm{rxn}}=-{\left(\frac{\delta \Delta G_{\mathrm{cell}}}{\delta T}\right)}_{P,N}=nF{\left(\frac{\delta E_{\mathrm{OCV}}}{\delta T}\right)}_{P,N}$$ 1

where *E*_OCV_ is equilibrium open-circuit voltage (OCV), *n* is the number of reacting electrons, and *F* is the Faraday constant. The Δ*S*_rxn_, which can be measured through isothermal potentiometric entropy measurements, in turn determines the reversible heat generation during battery operation, $\mathrm{Q̇}=IT\frac{\Delta S_{\mathrm{rxn}}}{nF}$ .^19,21–23^ Isothermal potentiometric entropy measurements have also helped determine the entropy of battery electrodes, which changes over their states of charge (SOC).^20,24–27^ These entropy measurements provide characterization of electrode intercalation phases and their state-of-health over time and operation.^19,20,28–31^ Despite the wealth of information provided by isothermal potentiometric entropic experiments, these measurements cannot discern the entropy of lithium-ion (de)­solvation at the electrode/electrolyte interface,^9^ as the (de)­solvation processes occur symmetrically at each interface under isothermal temperatures.

Nonisothermal, temperature gradient potentiometric measurements on symmetric-electrode cells, such as symmetric-lithium–metal-electrode cells, are instead used to probe the entropy of the solvation-desolvation processes in electrolytes. In these experiments, different temperatures are applied to each electrode of the symmetric cell, which asymmetrically alters each individual electrode’s potential.^32,33^ The resulting cell voltage change as a function of applied temperature gradient provides a $\frac{dE_{\mathrm{OCV}}}{dT}$ , referred to as the temperature coefficient for the remainder of this study. The measured temperature coefficient provides insight into factors that affect the entropy of the lithium-ion solvation-desolvation process in the cell, such as the ion-solvation/ion-coordination environment.^1,9^ The solvation environment, which is probed by these temperature gradients and determined by choice of salt, salt concentration, and solvent, can play a large role in the reaction pathway and kinetics of electrode reactions.^2^

In this study, we use potentiometric temperature coefficient measurements in a custom temperature gradient setup to determine the partial molar entropy of lithium-ion (de)­solvation in two commonly used solid electrolytes: sulfide-type argyrodite Li_6_PS_5_Cl (LPSCl) and oxide-type tantalum-doped garnet Li_6.5_La_3_Zr_1.5_T_0.5_O_12_ (LLZTO) solid-state electrolytes. A theoretical thermodynamic analysis of the electrochemical cell under a temperature gradient is first performed to characterize the potentiometric temperature gradient experiment. This analysis relates the observed temperature coefficients to the partial molar entropy of lithium ion solvation in a nonequilibrium environment. Through these experiments, we compare the distinct voltage responses of cells constructed with organic liquid electrolytes and solid-state electrolytes to applied temperature gradients. This analysis is extended to temperature coefficient measurements on a composite solid–liquid electrolyte system, providing a deeper understanding of temperature coefficients in more complex systems. The measured partial molar entropies are then used as a design guide for the temperature coefficient of composite cells with various cell properties, such as electrolyte thicknesses, thermal conductivities, and partial molar entropies of lithium ion solvation. These results serve as an initial characterization of the lithium ion “solvation” entropic properties of solid-state electrolytes while furthering our understanding of the temperature coefficient’s thermodynamic and electrochemical basis, as well as examine the application of these entropic properties to the design of cells.

Existing potentiometric analyses based on eq 1 only apply to equilibrium measurements because its derivation is based on Gibbs Free Energy, an equilibrium quantity. Therefore, to accurately relate temperature coefficients of symmetric lithium cells to the partial molar entropy of lithium ion solvation in these experiments, a nonequilibrium thermodynamic model based on electrochemical potentials is required. We begin by mapping out symmetric-cell components and temperatures, where the red color corresponds to higher temperatures and the blue color corresponds to lower temperatures (Figure 1A,B). The electrochemical potentials of the lithium ions, lithium–metal atoms, and electrons in the system are then assigned to their respective cell components and temperatures. For example, the lithium electrode on the hot end, noted as the “hot lithium electrode”, contains both electrons and lithium atoms at the hot temperature. Therefore, the hot lithium electrode has two associated electrochemical potentials, μ_e^–^,H_^E^ and μ_Li,H_^E^. Similarly, the electrochemical potential of the terminal that connects the electrode to the potentiostat only contains electrons at the hot temperature, μ_e^–^,H_^T^. A temperature gradient exists across the thickness of the solid electrolyte. As a result, the electrochemical potential of lithium ions in the solid electrolyte at the hot electrode interface, μ_Li^+^,H_^S^, differs from the lithium ions in the solid electrolyte at the cold electrode interface, μ_Li^+^,C_^S^. At both hot and cold electrode–electrolyte interfaces, there are lithium ion reduction reactions, Li_s_^+^ + e_E_^–^ *⇌*Li_E_ occurring at different temperatures. These two lithium ion redox reactions are used to find the cell potential. At a given constant temperature gradient applied, the system will achieve a steady state. At steady state conditions and assuming local equilibrium, the reactant and product electrochemical potentials for both hot-interface and cold-interface lithium-ion redox reactions are equal (Figure 1B, details in Supporting Information section S.1): μ_Li^+^,H_^S^ + μ_e^–^,H_^E^ = μ_Li,H_^E^ and μ_Li,C_^E^ = μ_Li^+^,C_^S^ + μ_e^–^,C_^E^.

**Figure 1:**
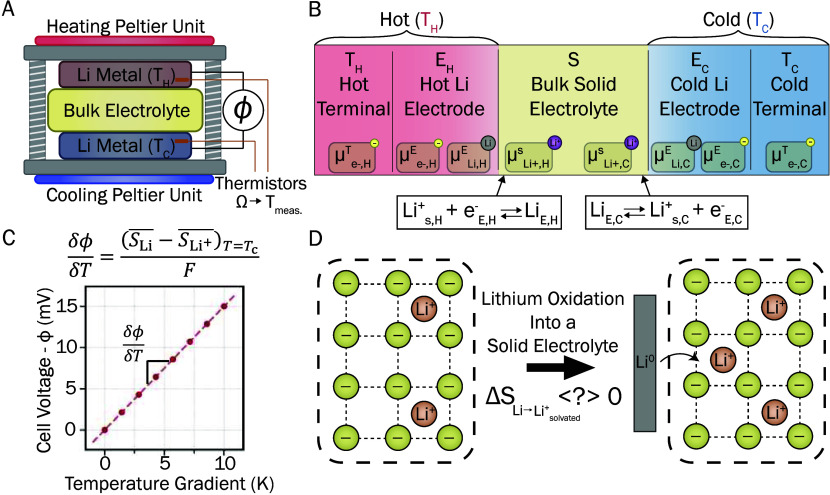
Using temperature gradients to investigate lithium-ion solid-state electrolyte solvation entropies. (A) Schematic of the setup used to measure the temperature coefficient of a symmetric lithium–metal cell with a given electrolyte system. (B) Interface model maps out the relevant electrochemical potentials, reactions, and temperatures in the cell. (C) Equation and example of the temperature coefficient in an example, fictional OCV-TG graph. (D) Interrogation of lithium-ion solvation entropy sign in solid-state electrolytes.

With these equalities, we can then expand out the electrochemical potentials. By assuming a negligible potential drop across the electrolyte (at open-circuit conditions) and expressing the hot lithium-atom and hot lithium-ion chemical potentials with reference to their cold counterparts through a Taylor expansion, we find

$$\frac{\delta \phi }{\delta T}=\frac{\left(\overset{®}{S_{\mathrm{Li}}\;}-\overset{®}{S_{{\mathrm{Li}}^{+}}\;}\right){|}_{T=T_{C}}}{F}$$ 2

where δ represents a difference in state (not a differential), ϕ is the cell voltage, *T* is temperature, $\overset{®}{S_{\mathrm{Li}}\;}$ is the partial molar entropy of lithium addition into the lithium–metal electrode, and $\overset{®}{S_{{\mathrm{Li}}^{+}}\;}$ is the partial molar entropy of lithium-ion addition into the electrolyte. The derivation of eq 2 is detailed in the Supporting Information (Section S.1). Equation 2 assumes that the partial molar entropies are constant over the temperature range tested, which was confirmed by ramping the overall temperature of a Li|LPSCl|Li cell under a constant three-degree gradient and observing nearly constant open-circuit voltage (Figure S1).

As a note, we assume that the temperatures across each electrode and terminals, which are fully metallic, are at equal temperatures such that temperature varies only across the electrolyte. We believe this assumption to be valid based on the order-of-magnitude greater thermal conductivity of lithium of 84.8 W m^–1^ K^–1^,^34^ in comparison to the solid electrolytes used, with LPSCl having *k*_LPSCl_ ∼ 0.5–0.65 W m^–1^ K^–1^,^35,36^ and LLZTO having *k*_LLZTO_ ∼ 1.2–1.4 W m^–1^ K^–1^.^35,37^

Equation 2 quantifies the relationship between the temperature coefficient $\frac{\delta \phi }{\delta T}$ and the partial molar entropies of lithium atoms for the lithium–metal electrode and lithium-ion “solvation” into/from the electrolyte (numerator on the right-hand side, respectively). Derivations from various methods for the single electrolyte case describe similar relationships.^38,39^ The (de)­solvation process in solid electrolytes can be more accurately referred to as (de)­intercalation, but we will keep “(de)­solvation” for the purposes of comparing solid and liquid electrolytes. Overall, by applying temperature gradients to the constructed cells, measuring resulting changes in cell voltage, and fitting a linear slope, we can measure the temperature coefficient (Figure 1A,C). Then by using eq 2, we explicitly solve for the partial molar entropy of lithium-ion solvation in the electrolyte, given that the partial molar entropy of lithium atoms for lithium metal is $\overset{®}{S_{\mathrm{Li}}\;}=29.1$ J mol^–1^K^–1^.^40,41^ These calculations will show whether lithium-ion “solvation” in the solid electrolyte is an entropically positive or negative process (Figure 1D). We can further consider the effect of a temperature change on the lithium-ion redox reaction equilibrium at a given electrode–electrolyte interface. If the partial molar entropy of lithium-ion solvation into the electrolyte is greater than the partial molar entropy of lithium atom addition to the lithium electrode $(\overset{®}{S_{{\mathrm{Li}}^{+}}\;}>\overset{®}{S_{\mathrm{Li}}\;}$ and $\frac{\delta \phi }{\delta T}<0)$ , increasing temperatures will raise the electrochemical potential of lithium atoms in the electrode relative to the electrochemical potential of solvated lithium ion in the electrolyte, shifting the reaction equilibrium toward lithium-ion oxidation from the electrode into the electrolyte. In contrast, if the partial molar entropy of lithium-ion solvation into the electrolyte is less than the partial molar entropy of lithium atom addition to the lithium electrode $(\overset{®}{S_{{\mathrm{Li}}^{+}}\;}<\overset{®}{S_{\mathrm{Li}}\;}$ and $\frac{\delta \phi }{\delta T}>0)$ , increasing temperatures will lower the electrochemical potential of lithium atoms in the electrode relative to the electrochemical potential of lithium ion in the electrolyte, shifting the reaction equilibrium toward lithium ion reduction out of the electrolyte and into the electrode.

With the relationship between the temperature coefficient and component partial molar entropies derived, a custom cell setup was fabricated to apply controlled temperature gradients to a symmetric Li|solid electrolyte|Li cell under realistic stack pressure conditions (Figure 1A, details in the methods). The cell setup was verified against existing measurements on 1 M LiPF_6_ in 1:1 vol % EC:DEC liquid electrolytes, with temperature coefficients ranging between 1.12 mV K^–1^,^33^ and 1.14 mV K^–1^.^9^ In comparison, our constructed cell exhibited a temperature coefficient of 1.09 ± 0.05 mV K^–1^ (Figure S2), confirming the accuracy of the setup for measuring temperature coefficients.

Using the verified setup, symmetric-cell voltages for the LPSCl and LLZTO solid electrolytes were measured under applied temperature gradients ranging from 0 to 20 K for the LPSCl cells and 0 to 10 K for the LLZTO cells (Figure 2A,B). A smaller temperature range was used for the LLZTO cells due to LLZTO’s greater thermal conductivity (1.59 W m^–1^ K ^–1^ ^42^ versus 0.5 W m^–1^ K^–1^ for LPSCl^35,36^ ), which lowered the achievable temperature gradient by the setup when the heating Peltier unit is operated close to its maximum capability. The cell voltage decreases with increasing applied temperature gradient for the LPSCl solid electrolyte (−0.147 mV K^–1^), whereas cell voltage increases with increasing applied temperature gradient for the LLZTO solid electrolyte (+0.028 mV K^–1^) (Figure 2A–D). The decrease in open-circuit voltage for the LPSCl cell with an increasing temperature gradient indicates that at lithium-agryodite interfaces, increasing temperatures shift the lithium-ion redox reaction toward lithium-ion oxidation into the LPSCl electrolyte. In contrast, the (small) increase in open-circuit voltage for LLZTO with increasing temperature gradient indicates that at lithium-garnet interfaces, increasing temperatures (slightly) shift the lithium-ion redox reactions toward lithium-ion reduction into the lithium–metal electrode. Negligible hysteresis (∼0.014 mV) is observed between the warming and cooling ramps for LPSCl (Figure 2A), indicating temperature history-independent measurements and measurements unaffected by bulk/grain boundary resistance growth possibly induced by elevated temperatures applied during the experiment (Figure S3B,E). Minor hysteresis (∼0.023 mV) is visually observed for LLZTO (Figure 2B). While the origin of this hysteresis is unknown, its presence may appear more pronounced by the small voltage response to the applied temperature gradient, which causes a reduced signal-to-noise ratio. The temperature coefficient’s pressure dependence was also evaluated by measuring the temperature coefficient of LPSCl cells under two times the operating pressure (Figure S4). The results verify the temperature coefficient’s independence of the absolute value of the pressure, as shown in eq 2.

**Figure 2:**
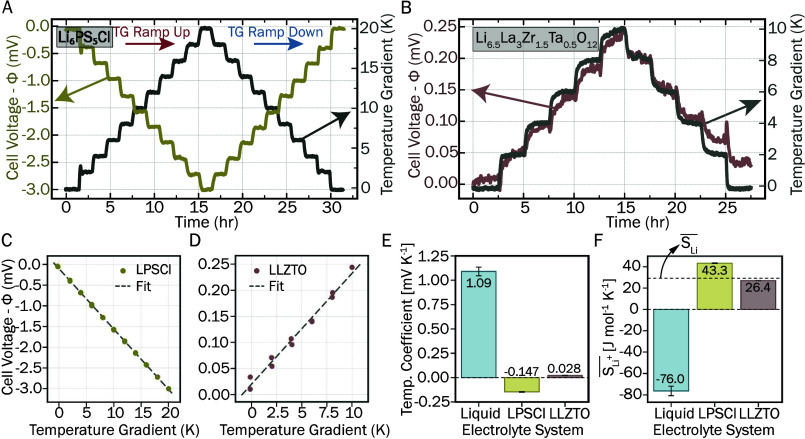
Cell open-circuit voltage and temperature data used to calculate solid electrolyte partial molar entropies. OCV and temperature gradient versus time graph of an example (A) Li_6_PS_5_Cl solid electrolyte Li-symmetric cell and (B) Li_6.5_La_3_Zr_1.5_T_0.5_O_12_ solid electrolyte Li-symmetric cell. Cell voltage versus temperature gradient for the (C) LPSCl cell and (D) LLZTO cell. (E) Bar chart summarizing the temperature coefficients of a 1 M LiPF_6_ EC:DEC liquid electrolyte system compared to the LPSCl and LLZTO systems. For each category, three samples were measured, with the sample standard deviation represented by the error bars. (F) Bar chart translates the results of the temperature coefficients and their corresponding standard deviations into partial molar entropies of lithium-ion solvation.

The measured temperature coefficients of lithium–metal symmetric cells with varying electrolytes were collected, summarized, and compared (Figure 2E). The temperature coefficient of the liquid electrolyte system (1 M LiPF_6_ in 1:1 vol % EC:DEC) is positive and large. In contrast, the LPSCl system has a negative temperature coefficient that is nearly an order-of-magnitude smaller, while the LLZTO system has a positive temperature coefficient even smaller in magnitude. The very small magnitude of the temperature coefficient in the LLZTO system means that additional effects on the temperature coefficient that are typically neglected in these potentiometric temperature gradient measurements, such as the Thomson effect (∼0.01 mV K^–1^)^1,43^ and the Soret effect (generally reported as <0.05 mV K^–1^)^1,44^ may appreciably contribute to the observed temperature coefficient. Though these errors may affect the measured value, it is important to note that the Soret effect in solid electrolytes is an unknown quantity. It is also important to note that, even if the measured temperature coefficient for LLZTO is maximally altered by these other effects, the ranges of values introduced by the error lead to the same implications on the sign of the partial molar entropy of lithium-ion solvation, as discussed next.

These measured temperature coefficients were translated to partial molar entropy via eq 2. The partial molar entropy of the liquid system is strongly negative while both solid electrolyte systems show positive partial molar entropies. The divergence in sign is hypothesized to be due to the fundamental differences in lithium-ion solvation mechanisms between liquid and solid electrolytes. When a lithium-ion is solvated by the liquid electrolyte, there is a corresponding loss in the degrees of freedom and entropy exhibited by the solvent molecules in the solvation shell.^7,45^ Therefore, these potentiometric measurements, and previously performed calorimetric measurements that measure reversible Peltier heat generation upon current application,^7^ examine the extent to which electrolyte composition (solvents, salts, concentrations, etc.) alter the magnitude of entropy decrease upon lithium-ion solvation in liquid electrolytes. In contrast to liquid electrolytes, solid-state electrolytes possess a rigid anionic framework through which lithium-ions diffuse or hop between stable/metastable sites (Figure 1D).^46^ This rigid anionic framework presumably suppresses the large translational/rotational entropy change associated with solvation shell formation in liquid electrolytes. The experimental results show that the “solvation” of lithium-ions into the solid-electrolytes is a positive-entropy process, potentially due to an increase in configurational entropy within the solid electrolyte upon the addition of a lithium-ion. To evaluate this hypothesis, we can assume a two-component ideal solution model in a solid electrolyte lattice where the two components are lithium-ion occupied sites and vacant lithium-ion sites. The chemical potential of the site-occupying lithium-ions is given by μ_Li^+^_ = μ_Li^+^_^o^ + *RT*ln­(*x*_Li^+^_),^47^ where *x*_Li^+^_ is the site occupation of lithium-ions. In LPSCl, which has a *F*4̅3*m* space group, there are 24 out of a total 72 possible lithium-ion sites stoichiometrically occupied.^48,49^ Similarly, in the undoped LLZO lattice, which has a *Ia*3̅*d* space group, 56 out of a total 120 possible lithium-ion sites are stoichiometrically occupied.^50^ By taking the derivative with respect to temperature, ${\left(\frac{\delta \mu_{{\mathrm{Li}}^{+}}}{\delta T}\right)}_{P,N_{i}}$ , and equating it to $-{\left(\frac{\delta S_{{\mathrm{Li}}^{+}}}{\delta N}\right)}_{{T,P,N}_{i\neq j}}$ via a Maxwell relation, we can find $\overset{®}{S_{{\mathrm{Li}}^{+}}\;}=-R\ln\left(x_{{\mathrm{Li}}^{+}}\right)$ . Using $x_{{\mathrm{Li}}^{+},\mathrm{LPSCl}}=\frac{24}{72}$ , $\overset{®}{S_{{\mathrm{Li}}^{+},\mathrm{LPSCl}}\;}=$ 9.13 J mol^–1^ K^–1^. Using $x_{{\mathrm{Li}}^{+},\mathrm{LLZO}}=\frac{56}{120}$ , $\overset{®}{S_{{\mathrm{Li}}^{+},\mathrm{LLZO}}\;}=$ 6.34 J mol^–1^ K^–1^. The positive values of $\overset{®}{S_{{\mathrm{Li}}^{+}}\;}$ capture the expected increase in configurational entropy of the solid-lattice based on the two-component ideal solution model. The smaller magnitudes of $\overset{®}{S_{{\mathrm{Li}}^{+}}\;}$ as calculated by this model versus the experimentally measured values, $\overset{®}{S_{{\mathrm{Li}}^{+},\mathrm{LPSCl}}\;}=$ 43.3 J mol^–1^ K^–1^ and $\overset{®}{S_{{\mathrm{Li}}^{+},\mathrm{LLZO}}\;}=$ 26.4 J mol^–1^ K^–1^, may reflect other contributions to the partial molar entropy of lithium-ion solvation in the solid-electrolyte lattice, such as changes in vibrational entropy.^20^ The gap between these experimental and theoretical partial molar entropy values can potentially be bridged through future computational studies.^17,51^

As an example of how the difference in partial molar entropy and thus the temperature coefficient between solid and liquid electrolytes would manifest in electrochemical cells, we revisit the work by Wang et al. that examined the underpotential lithium plating on graphite electrodes in the presence of an in-plane temperature gradient.^33^ The authors found that for a graphite electrode and lithium–metal counter electrode in a 1 M LiPF_6_ in 1:1 vol % EC:DEC liquid electrolyte (temperature coefficient of 1.12 mV K^–1^), a temperature gradient hotspot of ∼7 K greater than the surrounding regions can shift the equilibrium lithium reduction reaction potential above the room temperature lithium-ion graphite intercalation reaction, stated to be ∼10 mV in the room-temperature charged state. This shift of the lithium-ion redox reaction above the graphite intercalation reaction thermodynamically induces lithium plating on the graphite. Extending the presented workflow to the measured temperature coefficient/partial molar entropies for the LPSCl electrolyte (−0.147 mV K^–1^) and LLZTO electrolyte (0.028 mV K^–1^), we can calculate that a “cold spot” of around –68 K less than the surrounding regions would be needed for LPSCl and a hot spot of around 357 K greater than the surrounding regions would be needed for LLZTO to thermodynamically induce lithium plating on the graphite electrode. Both of these are unlikely temperature gradient conditions and indicate a lower thermodynamic susceptibility to the effects of temperature gradients in these cells.

The temperature coefficient analysis is extended to composite solid–liquid electrolytes, which consist of a core inorganic solid electrolyte sandwiched between two layers of liquid electrolyte that wet both the lithium metal electrode and the solid electrolyte. Practically, this architecture mitigates interfacial contact challenges between the electrode and the solid electrolyte by introducing a fluid, wetting liquid medium.^52–54^ By using a composite solid–liquid-electrolyte system, we can further investigate the fundamental origin of the temperature coefficient and its relation to the partial molar entropy of lithium-ion solvation while validating our previous temperature coefficient-to-solvation entropy measurements. The composite cell sandwiches an LPSCl solid electrolyte in between two identical layers of glass fiber separators soaked with 1 M LiPF_6_ in 1:1 vol % EC:DEC liquid electrolyte (Figure 3A). The lithium metal electrodes only contact liquid electrolyte. Four relevant interfaces with reactions are present in the cell, and four thermistors are assembled into the cell to measure these four temperatures and temperature drops across the cell. In this composite cell, beyond the previously considered lithium-redox reaction, there are additional lithium-ion transfers between the solid and liquid electrolyte so lithium-ions can traverse the cell. This transfer at the liquid electrolyte|solid electrolyte interface is modeled as a “transfer reaction”: ${\mathrm{Li}}_{S}^{+}⇌{\mathrm{Li}}_{l}^{+}$ (Figure 3B). As equilibrium redox reactions only occur at the lithium-metal|liquid-electrolyte interface (reaction 1), it is unclear whether adding solid electrolyte into the system and inducing the lithium-ion transfer process (reaction 2) affects the composite-cell temperature coefficient, which would be similar to the concept of the liquid–liquid electrolyte junction potential,^55^ or if only the lithium redox reactions play a role in determining the composite cell’s temperature coefficient.

**Figure 3:**
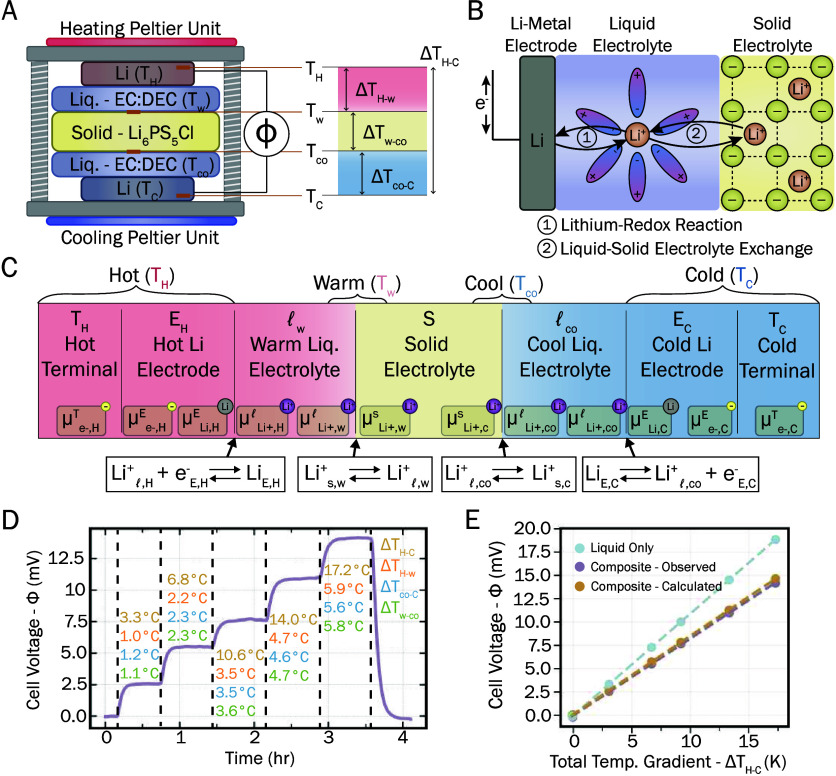
Evaluating the impact of a composite electrolyte on observed temperature coefficients. (A) Schematic of the setup used to measure the temperature coefficient for a composite solid–liquid-electrolyte system. (B) Two processes occur in the composite system, a lithium-ion redox reaction and a solid–liquid-electrolyte lithium-ion “solvation transfer reaction”. (C) Updated interface model for the composite solid–liquid-electrolyte system with “solvation transfer reactions”. (D) Composite-cell voltage and temperature gradient data for each step as a function of experiment time. (E) Cell-voltage as a function of total temperature gradient, Δ*T*_*H*–*C*_, for the composite cell in part D compared to a liquid-electrolyte-only cell and a theoretical composite cell predicted with eq 3.

The initial model used to interpret the single-electrolyte cell is expanded to describe the composite cell. As was performed in the single-electrolyte case, species’ electrochemical potentials are mapped out to represent the composite cell and relate the observed temperature coefficient to the species electrochemical potentials (Figure 3C). Modifying the interface model from the single electrolyte case, there are both solid- and liquid-electrolyte layers with distinct lithium ion electrochemical potentials: $\mu_{{\mathrm{Li}}^{+}}^{l}$ and μ_Li^+^_^s^. At steady state and assuming local equilibrium, there are four electrochemical potential equalities in the composite cell. Two equalities are associated with the lithium-ion redox reactions, $\mu_{{\mathrm{Li}}^{+},H}^{l}+\mu_{e^{-},H}^{E}=\mu_{\mathrm{Li},H}^{E}$ and $\mu_{\mathrm{Li},C}^{E}=\mu_{{\mathrm{Li}}^{+},C}^{l}+\mu_{e^{-},C}^{E}$ . Two equalities are associated with the lithium-ion “solvation transfer reactions” at the solid–liquid-electrolyte interface, $\mu_{{\mathrm{Li}}^{+},w}^{s}=\mu_{{\mathrm{Li}}^{+},w}^{l}$ and $\mu_{{\mathrm{Li}}^{+},\mathrm{co}}^{l}=\mu_{{\mathrm{Li}}^{+},\mathrm{co}}^{s}$ . The expansion of these electrochemical potentials results in

$$\phi =\frac{1}{F}\left[\Delta T_{\mathrm{H‐C}}\overset{®}{S_{\mathrm{Li}}\;}-\Delta T_{\mathrm{w‐co}}\overset{®}{S_{{\mathrm{Li}}^{+}}^{S}\;}-\left(\Delta T_{\mathrm{H‐w}}+\Delta T_{\mathrm{co‐C}}\right)\overset{®}{S_{{\mathrm{Li}}^{+}}^{l}\;}\right]$$ 3

where $\overset{®}{S_{{\mathrm{Li}}^{+}}^{S}\;}$ is the partial molar entropy of lithium-ions “solvating” into the solid electrolyte (LPSCl), $\overset{®}{S_{{\mathrm{Li}}^{+}}^{l}\;}$ is the partial molar entropy of lithium-ions solvating into the liquid electrolyte, Δ*T*_H–C_ is the temperature difference between the hot and cold lithium electrodes, Δ*T*_w‑co_ is the temperature drop across the solid electrolyte, Δ*T*_H‑w_ is the temperature drop across the liquid electrolyte on the hot side, and Δ*T*_co‑C_ is the temperature drop across the liquid electrolyte on the cold side. The derivation of eq 3 is detailed in the Supporting Information (Section S.2).

Composite cells were constructed, and voltage-temperature gradient measurements were performed (Figure 3D). Due to liquid electrolyte thermistor interference, manual temperature gradient ramps were performed (details in Methods). The data for the displayed composite cell is plotted and compared to a theoretical liquid-electrolyte-only cell and a theoretical composite cell as calculated by eq 3 using measured temperatures (Figure 3E). If only the lithium-ion redox reaction affects the cell voltage response, the observed cell voltage should closely follow the blue liquid electrolyte-only line because only liquid electrolyte contacts the lithium metal electrodes. Instead, the observed data shown in purple closely track the orange, calculated line. Therefore, the solid–liquid electrolyte transfer reaction does play a role in the cell voltage and thermodynamics of the cell and is well represented by the calculated eq 3 that includes both lithium-ion redox reactions and the lithium-ion transfer reaction.

As a component of this analysis, we note that the LPSCl solid electrolyte partial molar entropy changed when wetted with the 1 M LiPF_6_ in 1:1 vol % EC:DEC liquid electrolyte in this composite solid|liquid-electrolyte cell. The temperature coefficient and $\overset{®}{S_{{\mathrm{Li}}^{+}}^{S}\;}$ of the wetted LPSCl were measured in a separate Li|wetted LPSCl|Li symmetric cell to be 0.581 mV K^–1^ and –27.0 J mol K^–1^, respectively (Figures S5 and S6, details in Methods). This value is neatly between the $\overset{®}{S_{{\mathrm{Li}}^{+}}^{S}\;}$ of an as-synthesized LPSCl pellet and the $\overset{®}{S_{{\mathrm{Li}}^{+}}^{l}\;}$ of a 1 M LiPF_6_ in 1:1 vol % EC:DEC liquid electrolyte, and was used for all analyses where the LPSCl pellet was wetted. We assume that when the cell was rested under stack pressure, the degree of LPSCl wetting was consistent throughout the electrolyte, making a homogeneous, wetted LPSCl pellet with its own distinct properties from a liquid-only electrolyte layer or a solid-only electrolyte layer. Similar results were observed with a wetted LLZTO cell, where the temperature coefficient and partial molar entropy were measured to be 0.734 mV K^–1^ and – 41.7 J mol^–1^ K^–1^, respectively (Figures S6 and S7).

To apply the results of this temperature coefficient and partial molar entropy investigation to the electrochemical-thermal design of a composite cell, we incorporate the system thermal properties and thicknesses into eq 3 through one-dimensional heat-conduction analysis and arrive at a nondimensional relationship:

$$\frac{F}{RT_{C}}\frac{\phi }{\theta }=\overset{®}{S_{\mathrm{Li}}^{o}\;}\left[\frac{\left(1-L_{S}\right)\left(1-\overset{®}{S_{{\mathrm{Li}}^{+}}^{o,l}\;}\right)+\kappa L_{S}\left(1-\overset{®}{S_{Li^{+}}^{o,S}\;}\right)}{1-L_{S}+\kappa L_{S}}\right]$$ 4

where *R* is the gas constant, the nondimensional temperature difference is $\theta =\frac{T_{H}-T_{C}}{T_{C}}=\frac{\Delta T}{T_{C}}$ , the nondimensionalized solid-electrolyte length is $L_{S}=\frac{L_{S}}{L_{l,w}+L_{S}+L_{l,\mathrm{co}}}$ (where *L*_S_ is the solid electrolyte length, $L_{l,w}$ and $L_{l,\mathrm{co}}$ are the liquid electrolytes on the warm and cool side, respectively), the nondimensionalized thermal conductivity is $\kappa =\frac{k_{l}}{k_{S}}$ (where $k_{l}$ and *k*_S_ are the liquid and solid electrolyte thermal conductivities, respectively), the nondimensionalized lithium partial molar entropy is $\overset{®}{S_{\mathrm{Li}}^{o}\;}=\frac{\overset{®}{S_{\mathrm{Li}}\;}}{R}$ , the nondimensionalized liquid partial molar entropy is $\overset{®}{S_{{\mathrm{Li}}^{+}}^{o,l}\;}=\frac{\overset{®}{S_{{\mathrm{Li}}^{+}}^{l}\;}}{\overset{®}{S_{\mathrm{Li}}\;}}$ , and the nondimensionalized solid partial molar entropy is $\overset{®}{S_{{\mathrm{Li}}^{+}}^{o,S}\;}=\frac{\overset{®}{S_{{\mathrm{Li}}^{+}}^{S}\;}}{\overset{®}{S_{\mathrm{Li}}\;}}$ . The left-hand side of eq 4, $\frac{F}{RT_{C}}\frac{\phi }{\theta }$ , is the nondimensionalized temperature coefficient. Details for the derivation are provided in the Supporting Information (Sections S.3 and S.4)

This form of eq 4 eliminates the internal solid|liquid-electrolyte temperature measurements (*T*_w_ and *T*_co_ in Figure 3A), which would be difficult to measure in real systems. The nondimensional form further investigates the underlying system properties and ratios of properties, such as the ratio of electrolyte thicknesses and the ratio of solid-to-liquid electrolyte partial molar entropies. Furthermore, the liquid electrolyte lengths are encapsulated in the nondimensional solid electrolyte lengths, $L_{S}=1-L_{l,w}-L_{l,c}$ (details in Section S.4).

We then explore the cell design space to evaluate the equation’s ability to predict the effect of changing system properties on the temperature coefficient. We constructed composite cells of varying solid electrolyte pellet thicknesses and measured their temperature coefficients while comparing the measured nondimensional values against the expected nondimensional temperature coefficients (Figure 4A). The error shown is calculated using the uncertainties (sample standard deviations) in the solid and liquid partial molar entropy measurements. Orange points are observed temperature coefficients from fabricated composite cells, the blue point is the previously measured temperature coefficient of a liquid-electrolyte-only cell $\left(L_{S}=0\right)$ , the green point is the previously measured temperature coefficient of a wetted-solid-electrolyte-only cell $\left(L_{S}=1\right)$ and the gray line is the nondimensional temperature coefficient predicted via eq 4. An inset with the dimensional temperature coefficient as a function of pellet thickness is also included. As the pellet thickness increases, the relative temperature drop across the solid electrolyte increases in comparison to the temperature drops across the liquid electrolyte layers, lowering the cell temperature coefficient and thus lowering the overall cell voltage change in response to an applied temperature gradient to the electrodes. The composite cell temperature coefficients in orange generally follow the predicted temperature coefficients, showcasing the ability of eq 4 to predict the temperature coefficient of the composite cell with a given solid electrolyte thickness. We observe that varying degrees of solid-electrolyte wettedness (i.e., how much liquid electrolyte is used in the cell and how much liquid electrolyte is lost during cell fabrication) appear to alter the wetted solid-electrolyte partial molar entropy and thus affect the observed temperature coefficient. The values used in eq 4 are summarized in the Supporting Information (Table 3).

**Figure 4:**
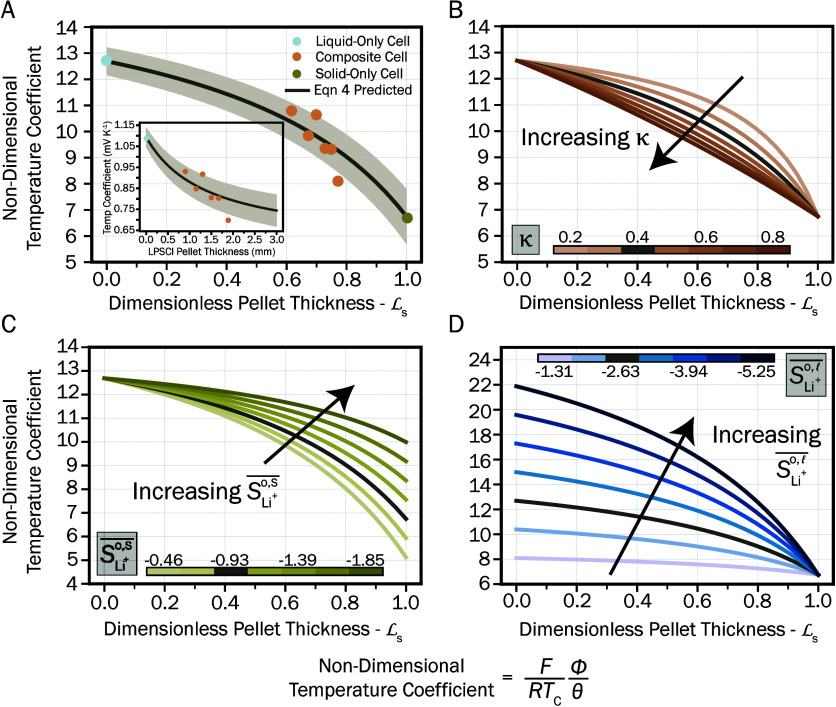
Describing composite cell system sensitivity to LPSCl pellet thickness changes. (A) Measured cell temperature coefficients as a function of solid electrolyte pellet thickness. (B) Shifts of the predicted temperature coefficients due to varying the magnitude of κ. The initial temperature coefficient and pellet thickness relationship is displayed in gray. (C) Shifts of the predicted temperature coefficients due to varying the magnitude of $\overset{®}{S_{{\mathrm{Li}}^{+}}^{o,S}\;}$ . (D) Shifts of the predicted temperature coefficients due to varying the magnitude of $\overset{®}{S_{{\mathrm{Li}}^{+}}^{o,l}\;}$ .

We also explore the impact of varying parameters of the cell components. First, we examine varying the nondimensional thermal conductivity parameter, κ, from 0.2 to 0.8 (κ_o_ = 0.4). An increase in κ would be achieved in practice by increasing the liquid electrolyte thermal conductivity or decreasing the solid electrolyte thermal conductivity. As κ increases, the rate of change of the temperature coefficient shifts at different $L_{S}$ (Figure 4B). However, the start and end points of the lines are unchanged due to the partial molar entropies of the solid and liquid electrolytes remaining unchanged. We can also examine the impact of choosing different solid and liquid electrolytes that have different partial molar entropies, leading to different $\overset{®}{S_{{\mathrm{Li}}^{+}}^{o,S}\;}$ and $\overset{®}{S_{{\mathrm{Li}}^{+}}^{o,l}\;}$ (Figure 4C,D). Shifting the electrolyte partial molar entropies will change the end points of the predicted temperature coefficients. As the magnitude of $\overset{®}{S_{{\mathrm{Li}}^{+}}^{o,S}\;}$ and $\overset{®}{S_{{\mathrm{Li}}^{+}}^{o,l}\;}$ increases (more negative), the predicted temperature coefficient lines shift upward. The increasing temperature coefficient lines reflect how increasing electrolyte partial molar entropies induce larger magnitude shifts of the lithium-ion redox reaction equilibrium toward lithium-ion reduction at the warm electrode.

In summary, lithium-ion partial molar entropies in two prominent lithium-conducting solid-state electrolytes, sulfide-type argyrodite Li_6_PS_5_Cl (LPSCl) and oxide-type tantalum-doped garnet Li_6.5_La_3_Zr_1.5_T_0.5_O_12_ (LLZTO), were measured using potentiometric temperature gradient measurements. Solid-state electrolytes exhibit positive lithium-ion partial molar entropies (43.3 J mol^–1^ K^–1^ for LPSCl and 26.4 J mol^–1^ K^–1^ for LLZTO) and organic liquid electrolytes exhibit negative lithium-ion partial molar entropies. Lithium symmetric cells fabricated with solid-state electrolytes also exhibit lower-magnitude temperature coefficients than lithium symmetric cells fabricated with organic liquid electrolytes. A one-dimensional analytical model for composite solid–liquid systems reveals that the temperature coefficient is governed by both lithium-ion redox reactions and lithium-ion transfer reactions between solid–liquid electrolyte interface, rather than being solely determined by electrode–electrolyte interactions. Overall, this work serves as an initial characterization of the thermal properties and entropy of lithium-ion solvation in solid-state cells, as well as a deeper study into the nature of the temperature coefficient measurement and its application to cell design.

## Methods

### Materials

Argyrodite “regular” lithium phosphorus sulfur chloride (Li_6_PS_5_Cl, LPSCl) powder of 3–5 μm particle size was purchased from NEI Corporation. As precursors for lithium lanthanum zirconium tantalum oxide (Li_6.5_La_3_Zr_1.5_T_0.5_O_12_, LLZTO), lithium hydroxide (LiOH) was purchased from Sigma-Aldrich, lanthanum oxide (La_2_O_3_) and zirconium oxide (ZrO_2_) were purchased from Acros Organics, and tantalum oxide (Ta_2_O_5_) was purchased from Alfa Aesar. LLZTO powder was synthesized using a ball milling and sintering procedure as outlined in our group’s previous work.^56^ Ethylene carbonate (EC), diethyl carbonate (DEC), and lithium phosphorus hexafluorophosphate salt were purchased from Capchem. Glass microfiber filters (Cytiva, Whatman GF/A) with a nominal thickness of 260 μm were used as the separator in liquid and composite cells. 200 μm thick lithium foil was purchased from Wellcos Corporation. NTC thermistors (Digikey, Sentec Corporation) are used for electrode temperature monitoring. Before incorporation into the electrochemical cells, the thermistor heads are wrapped in kapton tape. Electrical tape (3 M Scotch Super 33+) and air sealing tape (3 M Grid Air Sealing Tape) are used for cell sealing.

### Cell Construction

All cells were constructed in a 10 mm diameter PEEK plunger-type cell body with corresponding 10 mm diameter stainless steel plungers. The plungers are used to apply densification pressure and operating pressure as well as provide electrical contact to the electrodes. All cells were rested overnight after completion before potentiometric temperature gradient experiments were performed. The various cell configurations are summarized in the Supporting Information (Figure S9).

#### Solid-State Li|Electrolyte|Li Cell Construction

For Li|LPSCl|Li cells, 270 mg of the LPSCl powder is pressed into pellets in the plunger cell using a hydraulic press in an argon-filled glovebox at 400 MPa of pressure for 3 min.^57^ Thermistors used to measure electrode temperature are subsequently sandwiched between three 9 mm-diameter lithium discs and affixed to their respective plunger before inserting the plungers into the cell body. The extra lithium discs even out the bulge created by the thermistor head. The cells are then sealed using a layer of electrical tape and air sealing tape. After sealing, ∼ 2.8 MPa of operating pressure are applied. Screws are used as electrical leads to the cell plungers.

For Li|LLZTO|Li cells, 1.01 g of synthesized LLZTO powder is pressed into 12 mm-diameter pellets with a die set at ∼3.5 tons for 3 min, sintered at 1230 °C, polished, and washed in hexanes. Postsintering, the pellet is about 10 mm in diameter.^57,58^ In an argon-filled glovebox, one 9 mm-diameter lithium disc is affixed to either side of the LLZTO pellet and pressed using a cold-isostatic press (CIP, MSE Supplies) at 300 MPa for 45 min to achieve adequate interfacial contact. Each thermistor is then sandwiched between two additional 9 mm-diameter lithium discs and affixed to their respective plungers. Afterward, the Li|LLZTO|Li assembly is placed inside the cell body, and the plungers are inserted. The cell is then sealed with a layer of electrical tape and air sealing tape. After sealing, ∼2.8 MPa of operating pressure are applied.

#### Composite Solid–Liquid and Liquid-State Li|Electrolyte|Li Cell Construction

For both the liquid and solid–liquid composite, 1 M LiPF_6_ in 1:1 vol % EC:DEC was used. LiPF6 was measured and dissolved in the EC:DEC mixture under stirring overnight to form a 1 M solution. The Whatman glass microfiber separator was punched into 10 mm discs and vacuum oven-dried overnight. In both liquid and composite setups, each thermistor was sandwiched between three 9 mm-diameter lithium discs and affixed to their respective plunger. All fabrications were performed inside an argon-filled glovebox.

For the Li|EC:DEC|Li setup, 6 glass microfiber pieces were stacked in the cell body to provide adequate thermal resistance and create a temperature gradient between the two lithium electrodes. 100 μL of electrolyte was used to soak the glass microfiber pieces. Then, the plungers with lithium electrodes and thermistors were inserted into the cell body and the cell was sealed with a layer of electrical tape and air-sealing tape. After sealing, ∼2.8 MPa of operating pressure are applied. Although the pressure is unnecessary for maintaining contact at the lithium-liquid electrolyte interface, the pressure ensures that the tape can adequately seal the cell while also ensuring sufficient contact between the several lithium layers used.

For the composite Li|EC:DEC|LPSCl|EC:DEC|Li setup, varying amounts of LPSCl powder, depending on the desired pellet thickness, were pressed into a pellet at 400 MPa for 3 min. After pellet fabrication, a thermistor was placed on the pellet and covered with four layers of glass microfiber on both sides of the LPSCl pellet. The glass microfiber separator pieces were then soaked with 60 μL of electrolyte on each side, with small adjustments in the volume of liquid added based on the LPSCl thickness. The plungers with lithium electrodes and thermistors were subsequently inserted into the cell body, and the cell was sealed with a layer of electrical tape and air-sealing tape. ∼2.8 MPa of operating pressure was applied for the same reasons as mentioned when fabricating the liquid-electrolyte-only cell. For the composite Li|EC:DEC|LLZTO|EC:DEC|Li setup, the same procedure was followed with a LLZTO pellet, with 45 μL of electrolyte on each side.

For Li|wetted-LPSCl|Li cells and Li|wetted-LLZTO|Li cells, the same LPSCl and LLZTO pellet procedures are followed as previously described. One layer of Celgard 2320 is subsequently added to each side of the pellet to contain the liquid electrolyte within the cell, and 20 μL of electrolyte is dropped on both sides. This layer of Celgard is considered to be thin enough to be treated as a layer of liquid with negligible thickness and thermal resistance, which makes it distinct from the composite solid–liquid cell, which has several layers of thicker glass fiber and liquid electrolyte. We also note that wetted-LPSCl cells without Celgard 2320 show similar initial measurements, but latter portions of experiments showed signs of drying out. The plungers with lithium electrodes and thermistors were then inserted, the cell was sealed with a layer of electrical and air-sealing tape, and ∼2.8 MPa of operating pressure was applied.

### Temperature Coefficient and Electrochemical Measurements

Open-circuit voltage measurements were taken in 10 s intervals with a resolution of 50 μV, using a VMP-3 potentiostat (Biologic). Triplicate measurements were taken for each temperature coefficient measurement of the solid-state electrolyte symmetric cells, organic liquid electrolyte symmetric cells, and the wetted LPSCl symmetric cells. For solid-state cells with feedback-controlled temperature gradient steps, the voltage and temperature gradient measurements were averaged over the last 60 min of each step. For liquid and composite cells with manually controlled temperature gradient steps, the voltage and temperature gradient measurements were averaged over the last 5 min of each step.

Electrochemical impedance spectroscopy (EIS) measurements were performed using an SP-300 potentiostat (Biologic) to evaluate degradation during the temperature coefficient measurements. All-solid-state cells and the wetted LPSCl cells were scanned from 7 MHz to 1 Hz with 7 points per decade and 10 measures per frequency. Liquid-electrolyte cells were scanned from 250 kHz to 100 mHz with 10 points per decade and 2 measures per frequency. Solid–liquid-electrolyte composite cells were scanned from 7 MHz to 100 mHz at 7 points per decade and 5 measures per frequency. Fitting was performed on the EIS data using RelaxIS 3 software, with equivalent-circuit models discussed below. Parallel (R)­(P) circuit component capacitances were calculated using the RelaxIS electrochemical calculator tool based on fitted parameters to assign the (R)­(P) components to cell processes (where R represents a resistor and P represents a constant-phase-element). Liquid-only cell EIS data were fitted to an equivalent circuit model assuming the semicircle represented an interfacial process. Solid-only cell EIS data were fitted to an equivalent circuit model from Singh et al. for a symmetric lithium–metal LPSCl cell, who assumed a single (R)­(P) element representing the bulk resistance.^58^ The capacitance for the (R)­(P) element in the pre-experiment EIS was 3.47 × 10^–11^ F, indicating a bulk transport resistance source.^58,59^ The composite solid–liquid cell was fitted to an equivalent circuit model adapted from Busche et al. for a symmetric lithium, composite liquid-LAGP 4-probe cell^60^ and Leng et al. for a symmetric LiFePO_4_ composite liquid-LLZTO H-cell.^52^ In both, one (R)­(P) element encapsulates the electrolyte bulk resistances, the second (R)­(P) element represents the solid electrolyte grain boundary resistance, and the third (R)­(P) element represents the solid–liquid interface resistance. Only two semicircles were resolved in the EIS for the composite liquid-LPSCl cell data, so they were represented with two (R)­(P) elements. Capacitances for the pre-experiment sample were calculated to be 2.41 × 10^–9^ F for the first (R)­(P) element and 8.46 × 10^–7^ F for the second (R)­(P) element. These were assigned to intergrain boundary resistance in the LPSCl solid electrolyte and solid–liquid phase boundary transfer resistance, respectively.^59,60^

### Temperature Gradient Control System

Peltier heatsink and fan assemblies (Adafruit) were modified to allow for pressure application to the assembled cells via a nut-and-screw system. The resistances of thermistors assembled into the cell were read via an Arduino unit and converted to measured temperatures. For the proportional-integral-derivative (PID) control system used in solid-state cells, measured temperatures were fed into a custom-programmed PID system that autonomously determined applied voltages to each Peltier unit to achieve the desired temperature (Figure S10A). Experiments were carried out for at most 1.5 days for the solid electrolyte experiments and less than 8 h for the liquid and composite cell measurements. For automated feedback control systems, all data for the experiment, including electrode temperatures, temperature gradient, and Peltier voltages/current/power, were recorded (Figure S11). For liquid and composite cells, the liquid electrolyte was found to interfere with thermistor measurements. If the OCV was being measured via potentiostat leads at the same time that current was running through the thermistor heads to measure their resistance, the potentiostat would read an altered OCV, while the Arduino unit would read unexpected, oscillating thermistor resistances. Therefore, for experiments with liquid electrolytes, the OCV and temperature measurements were manually altered over the course of a day. The cause of the interference is hypothesized to be due to electrical/electrochemical interactions between the thermistor head and its surrounding cell components, including the liquid electrolyte. Such interference during simultaneous temperature and OCV measurements is not observed in the cell fabricated with only a solid electrolyte, presumably because there is no liquid electrolyte seepage into the thermistor head or the environment surrounding the thermistor head that is typically protected by a layer of Kapton tape. In this hypothesis, the liquid electrolyte acts as the conduit for crosstalk between the thermistor head and potentiostat leads via the surrounding lithium metal electrodes. However, the exact nature of this interaction has not been clearly understood.This interference introduced by the liquid electrolytes may be a phenomenon that is associated only with this thermistor model/design and this particular electrochemical-cell setup.

## Supplementary Material



## Notes

Supporting figures and equation derivations (PDF)
