# Supplementary material for: Lithium-Ion Partial Molar Entropies in Liquid, Composite, and Solid-State Electrolytes
Source: J Phys Chem Lett. 2026 Jun 22;17(26):7454–64. doi: 10.1021/acs.jpclett.6c00870 (PMC13339763; doi:10.1021/acs.jpclett.6c00870)
Supplement: Supplementary file 1 [file jz6c00870_si_001.pdf]

# Supporting Information for Lithium-Ion Partial Molar Entropies in Liquid, Composite, and Solid-State Electrolytes

Austin Fan,[†,‡] Patrick J. West,[¶] Louis Vincent Morris,[¶,§] Dimitrios Fraggedakis,[‖] Rachel Carter,[⊥] and Kelsey B. Hatzell[*,#,‡]

†*Chemical and Biological Engineering, Princeton University, Princeton, NJ 08544, United States*

‡*Andlinger Center of Energy and the Environment, Princeton University, Princeton, NJ 08544, United States*

¶*Chemistry Division, Naval Research Laboratory, Washington, D.C., 20375, United States*

§*National Research Council Post-Doctoral Fellow*

‖*Department of Chemical and Biological Engineering, Princeton University, Princeton, NJ 08544, United States*

⊥*Department of Mechanical Engineering, University of Kansas, Lawrence, KS, 66045, United States*

#*Department of Mechanical and Aerospace Engineering, Princeton University, Princeton, NJ 08544, United States*

E-mail: kelsey.hatzell@princeton.edu

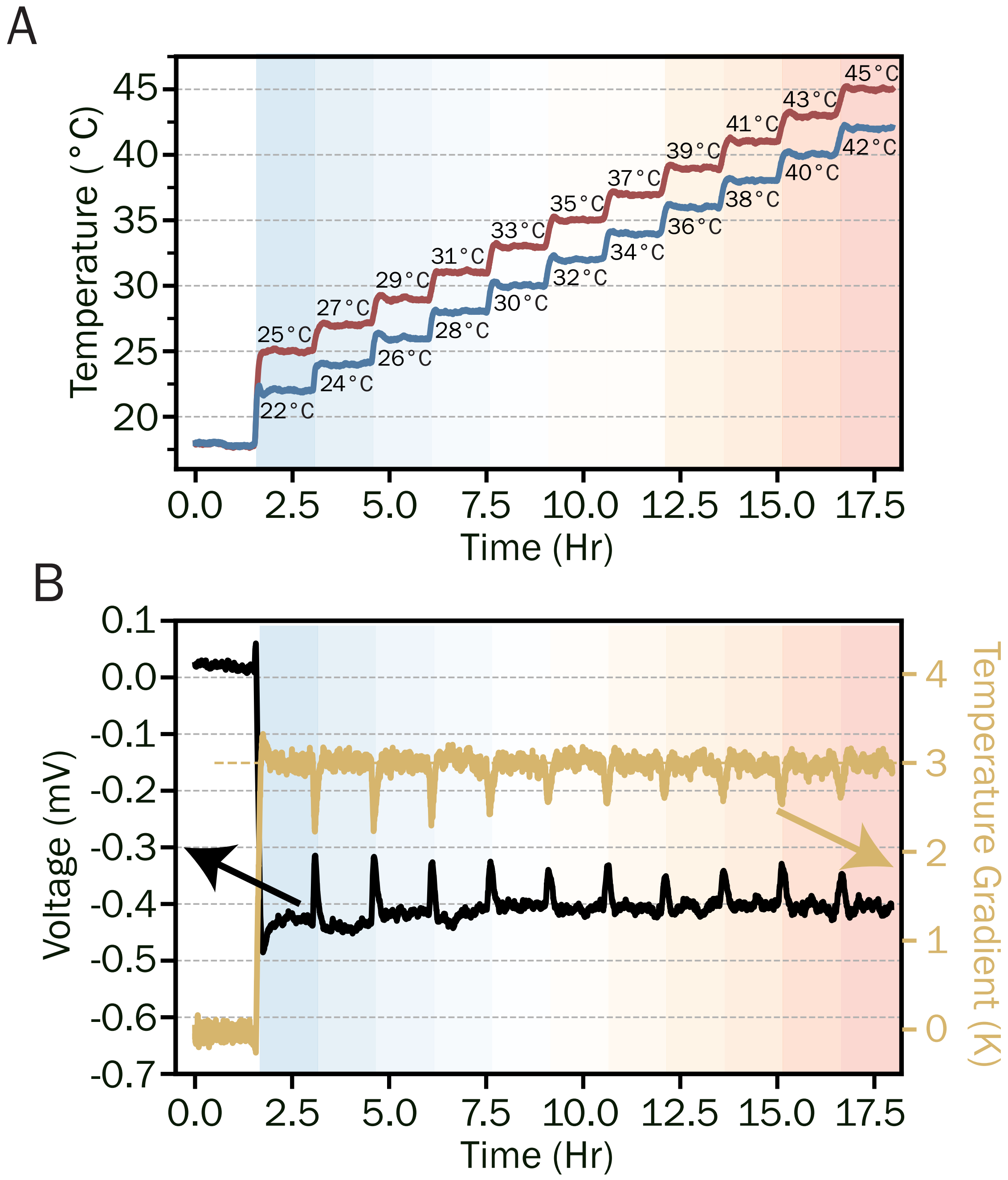


Figure S1: Constant Entropy Experiment. (A) A temperature ramp was performed on a symmetric Li|$Li_6PS_5Cl$|Li under a constant three degree temperature difference between the symmetric lithium electrodes. (B) The measured open-circuit voltage is nearly constant throughout the experiment, showing nearly constant entropy over the temperature range tested.

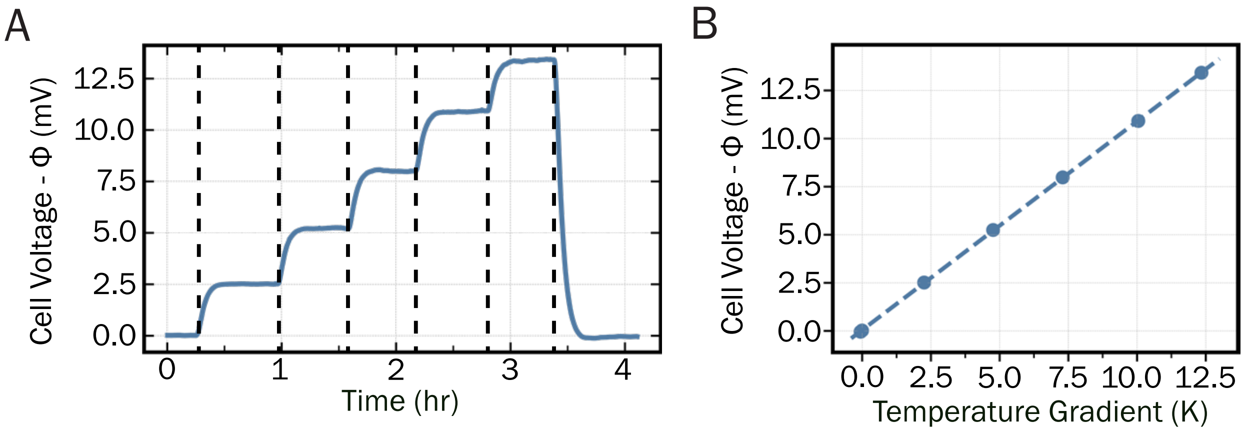


Figure S2: Liquid electrolyte, symmetric lithium-metal cell temperature gradient experiment. (A) Liquid cell voltage vs time experiment. (B) Liquid cell voltage vs. measured temperature gradient. Sample slope = 1.09 mV $K^{-1}$

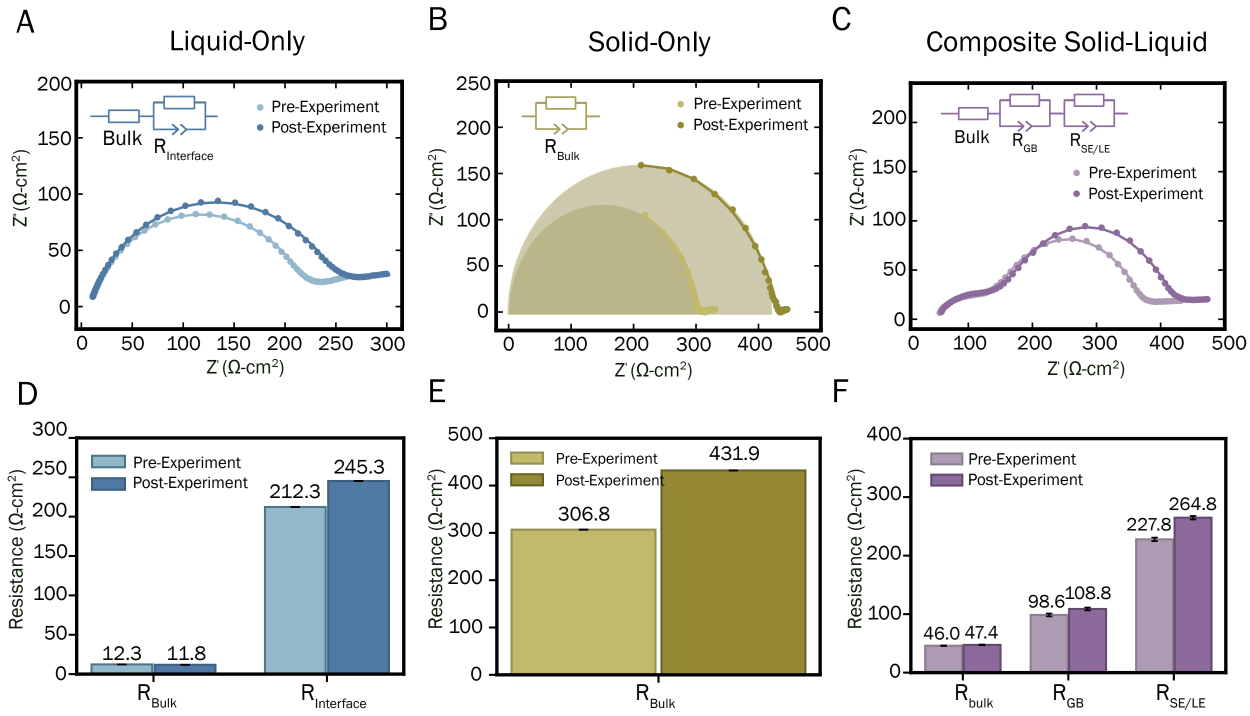


Figure S3: Electrochemical impedance spectroscopy for comparing pre- and post-experiment and corresponding (pre-test cell) parallel resistor-constant-phase-element circuit component capacitances in a (A) liquid-only cell ($C_1 = 7.2 * 10^{-7}$ F), (B) LPSCl cell ($C_1 = 3.47 * 10^{-11}$ F), and (C) composite solid-liquid cell ($C_1 = 2.41 * 10^{-9}$ F, $C_2 = 8.46 * 10^{-7}$ F). The impedance spectra for each were fitted to their respective equivalent circuit models shown in parts A-C for (D) liquid-only, (E) solid-only, and (F) composite solid-liquid. Error bars in D-F correspond to the error in fitting cell impedance data to the corresponding equivalent circuit model in RelaxIS.

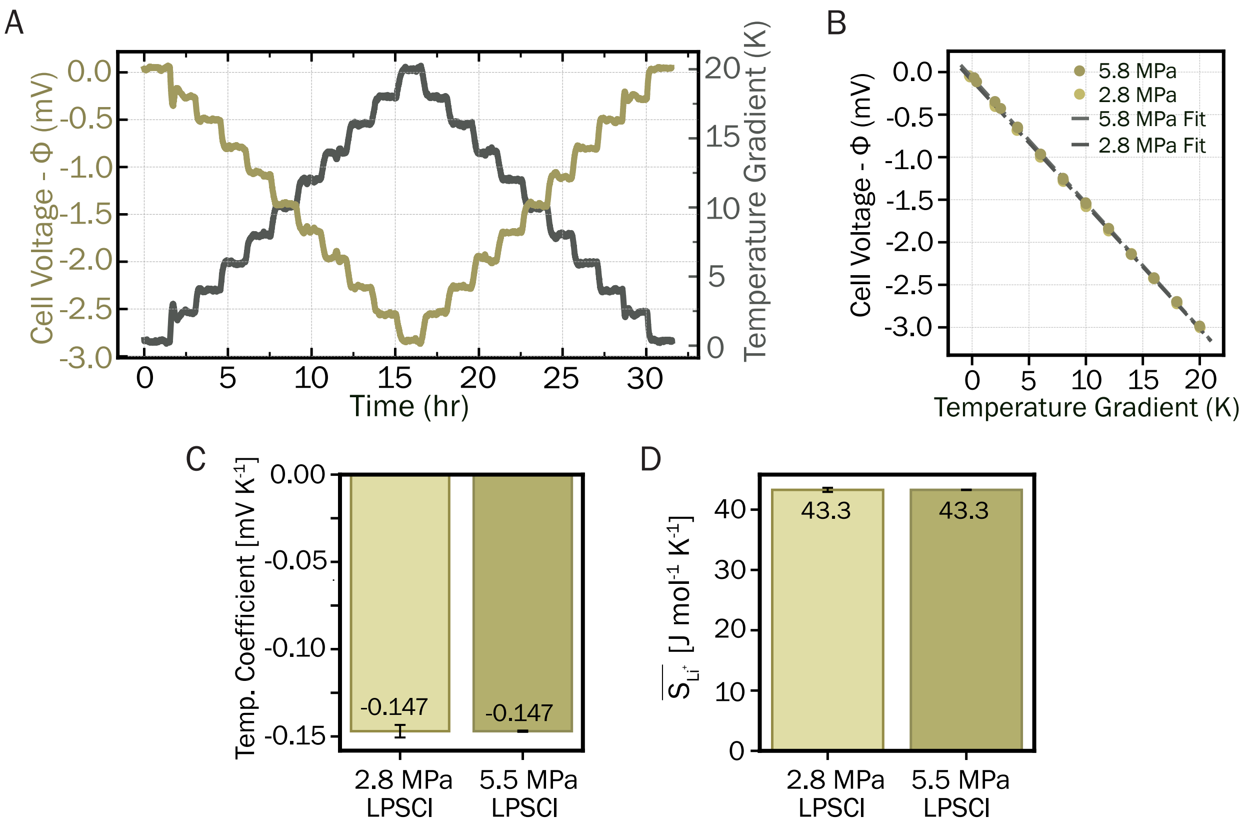


Figure S4: Temperature gradient experiment with a LPSCl cell under two times larger operating pressure. (A) Cell voltage, temperature gradient vs. time experiment. (B) Comparing voltage versus measured temperature gradient of a LPSCl cell voltage operated at $\sim$ 5.5 MPa and a LPSCl cell operated at $\sim$ 2.8 MPa. (C) Temperature coefficient of high- versus normal-pressure LPSCl cells. (D) Partial molar entropies of lithium-ion solvation of high- versus normal-pressure LPSCl cells. Two $\sim$ 5.5 MPa samples were measured.

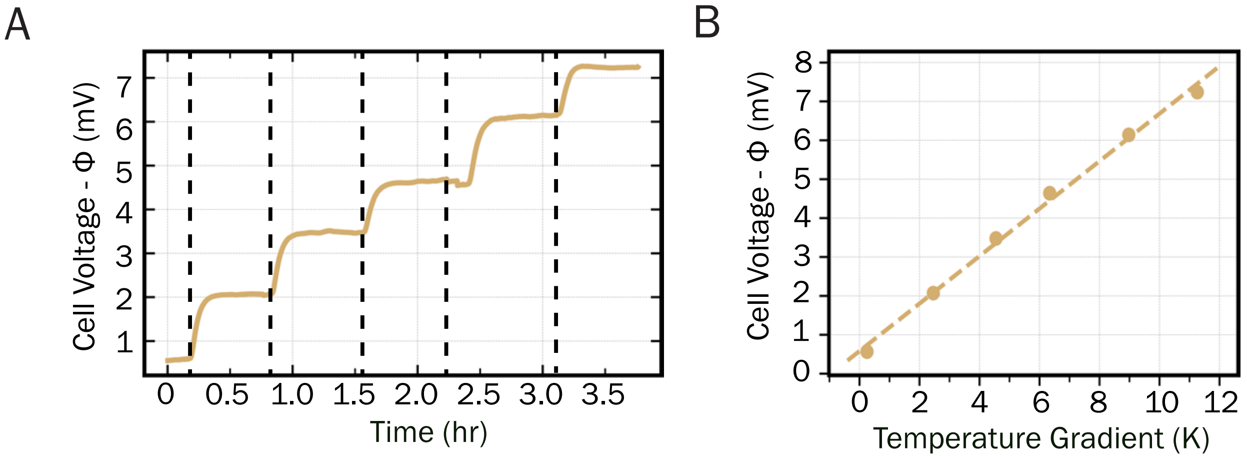


Figure S5: Example wetted LPSCl electrolyte, symmetric lithium-metal cell temperature gradient experiment. (A) Cell voltage vs. time experiment. (B) Cell voltage vs measured temperature gradient. Sample = 0.611 mV $K^{-1}$

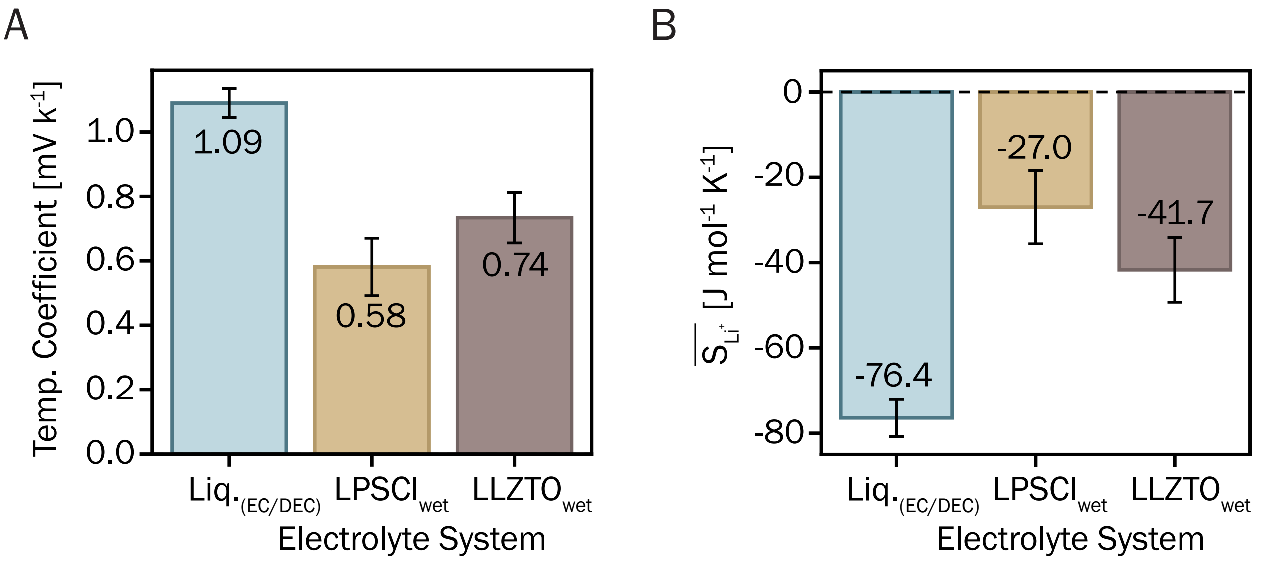


Figure S6: Liquid electrolyte compared to the wetted LPSCl and wetted LLZTO electrolyte. (A) The temperature coefficients of lithium symmetric-cells with either a liquid electrolyte, wetted LPSCl, or wetted LLZTO electrolyte. (B) Translated partial molar entropies of the liquid electrolyte, wetted LPSCl electrolyte, and wetted LLZTO electrolyte using eq. (2). For the liquid and wetted LPSCl systems, three samples were measured. For the wetted LLZTO system, two samples were measured. Error bars represent the sample standard deviation.

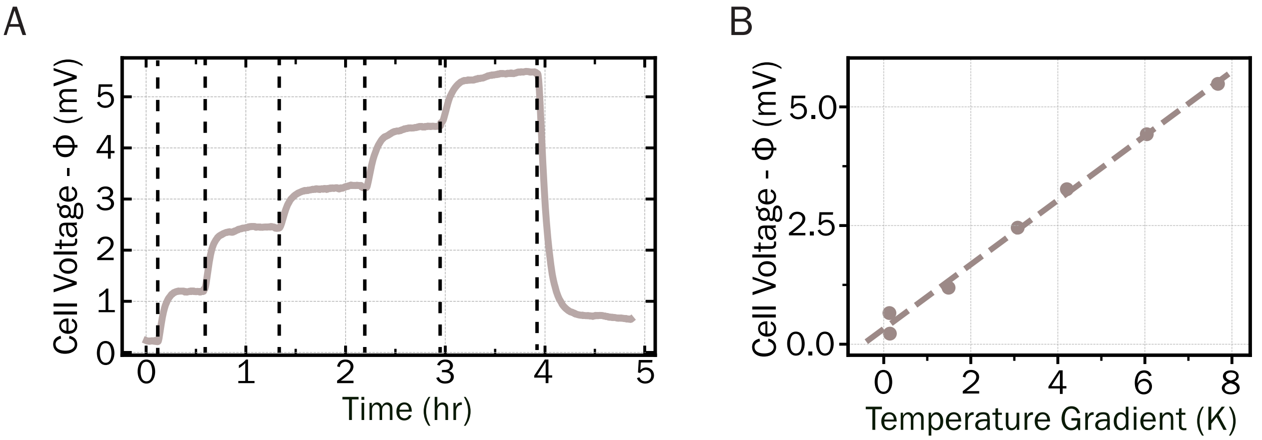


Figure S7: Example wetted LLZTO electrolyte, symmetric lithium-metal cell temperature gradient experiment. (A) Cell voltage vs. time experiment. (B) Cell voltage vs measured temperature gradient. Sample slope = 0.678 mV $K^{-1}$

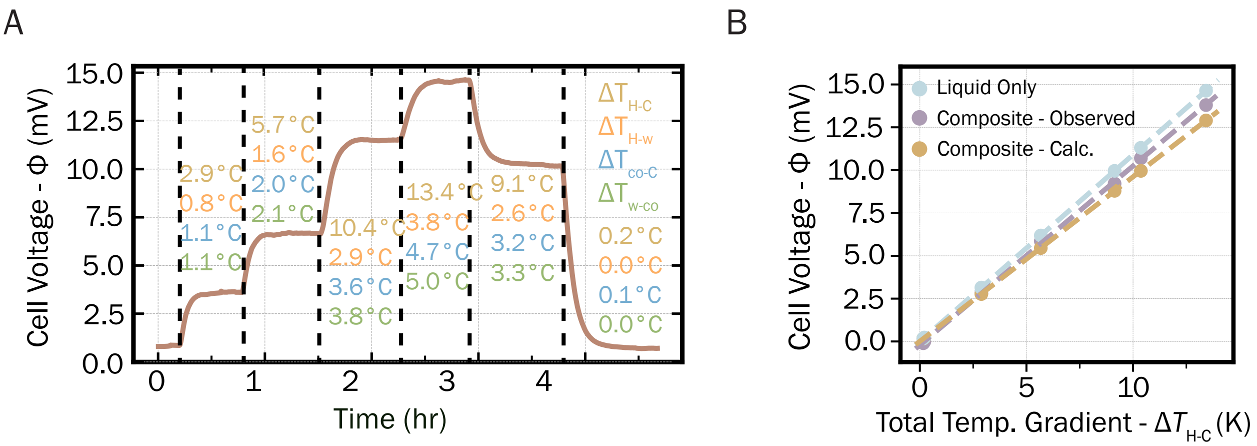


Figure S8: Composite solid-liquid LLZTO cell. (A) Cell voltage and temperature gradients vs. time data. (B) Cell-voltage as a function of total temperature gradient, $\Delta T_{H-C}$, for the composite solid-liquid LLZTO cell compared to a liquid-electrolyte-only cell and a theoretical composite cell predicted with eq. (3).

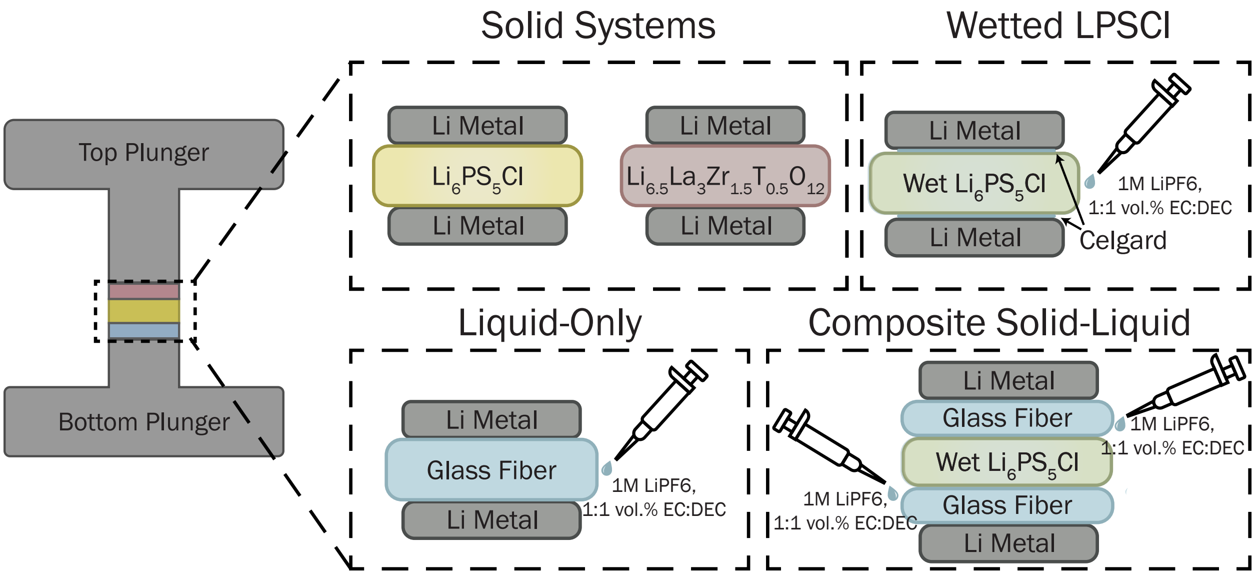


Figure S9: Lithium-symmetric cell constructions. Solid systems are constructed either using sulfide $Li_6PS_5Cl$ or oxide $Li_{6.5}La_3Zr_{1.5}T_{0.5}O_{12}$ solid electrolytes. A liquid-only system soaks the glass fiber piece in liquid electrolyte. The wetted-LPSCl system wets the LPSCl pellet with liquid electrolyte, and includes one layer of Celgard to help retain liquid while providing little thermal resistance. The composite solid-liquid system sandwiches an LPSCl pellet in between wetted glass fiber pieces.

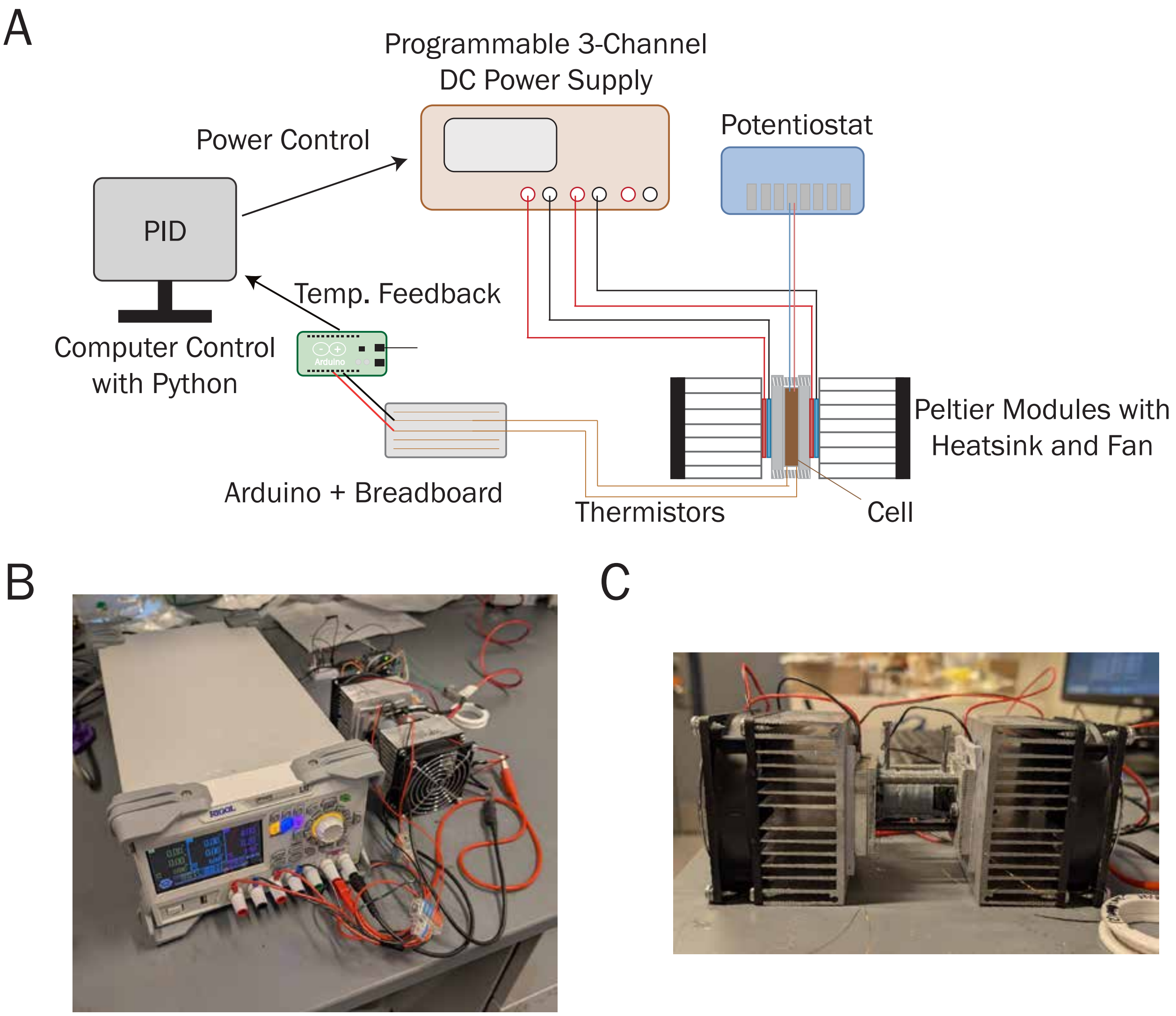


Figure S10: Cell setup. (A) Schematic of the cell feedback loop used to control a desired temperature gradient. (B) Image of cell setup with the power supply shown. (C) Cell configuration shown with the cell sandwiched in between two thermoelectric assemblies.

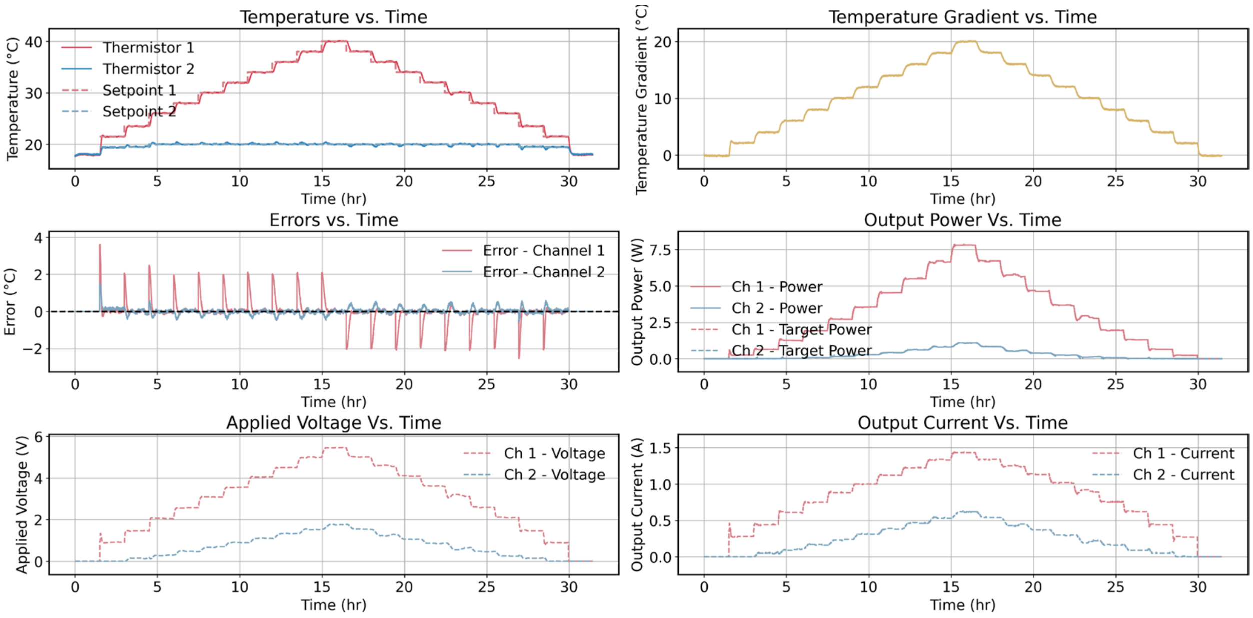


Figure S11: Recorded data for an example temperature gradient control experiment with a single electrolyte. The top left is the measured electrode temperatures, where thermistor 1 measures the hot-electrode temperature and thermistor 2 measures the cold-electrode temperature. The top right is the measured temperature gradient. The center left is the error between the electrode temperature and the temperature setpoint. During each temperature gradient step where the hot electrode temperature is changed by ±2 °C, the error between the electrode temperature and temperature setpoint will temporarily increase to ±2 °C. The center right is each peltier unit's output power, where channel 1 is the peltier unit applying temperature to the hot electrode while channel 2 is the peltier unit applying temperature to the cold electrode. The bottom left is the applied voltage to each peltier unit. The bottom right is the current through each peltier unit.

## S.1 Derivation of the Nonequilibrium Single-Electrolyte Partial Molar Entropy - Temperature Coefficient Relation

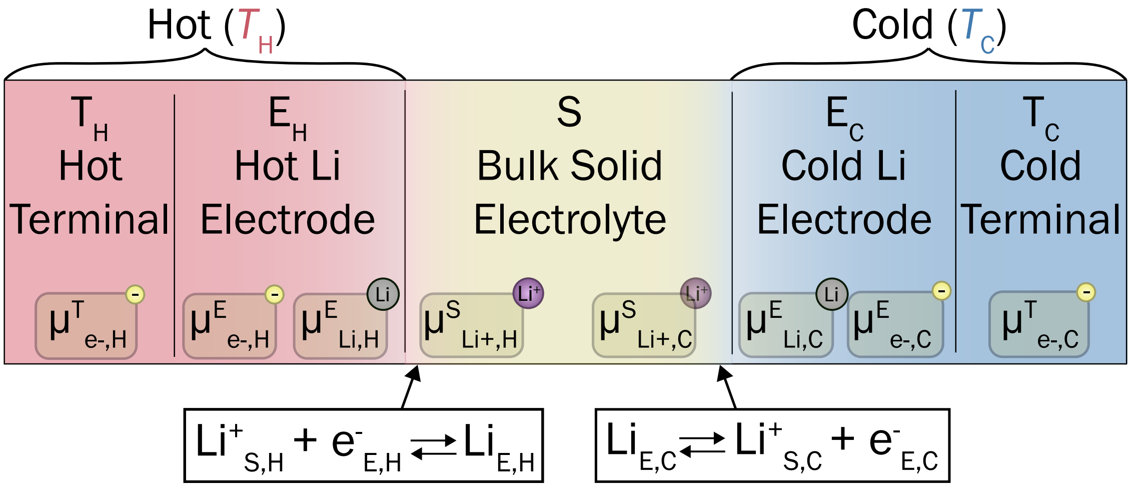


Figure S.1.1: Single Electrolyte Cell Interface Diagram

We begin from the mapped out lithium-ion, lithium-atom, and electron species electrochemical potentials at their respective temperatures where they are present in the symmetric cell (Fig. S.1.1). Red colors represent the hot side of the cell, and blue colors represent the cold side of the cell. Table I defines the electrochemical potential terms.

Table I: Electrochemical Potential Definitions

| Symbol | Definition |
|---|---|
| $\mu^{\mathrm{T}}_{\mathrm{e^-,H}}$ | $\mu$ of electrons in the hot terminal |
| $\mu^{\mathrm{T}}_{\mathrm{e^-,C}}$ | $\mu$ of electrons in the cold terminal |
| $\mu^{\mathrm{E}}_{\mathrm{e^-,H}}$ | $\mu$ of electrons in the hot lithium-metal electrode |
| $\mu^{\mathrm{E}}_{\mathrm{e^-,C}}$ | $\mu$ of electrons in the cold lithium-metal electrode |
| $\mu^{\mathrm{E}}_{\mathrm{Li,H}}$ | $\mu$ of lithium atoms in the hot lithium-metal electrode |
| $\mu^{\mathrm{E}}_{\mathrm{Li,C}}$ | $\mu$ of lithium atoms in the cold lithium-metal electrode |
| $\mu^{\mathrm{S}}_{\mathrm{Li^+,H}}$ | $\mu$ of lithium-ions at the solid electrolyte's $T_{\mathrm{H}}$ interface |
| $\mu^{\mathrm{S}}_{\mathrm{Li^+,C}}$ | $\mu$ of lithium-ions at the solid electrolyte's $T_{\mathrm{C}}$ interface |

We assume that the electron electrochemical potential does not appreciably change across the plungers used in the cell to apply stack pressure. Therefore, the plungers are omitted from the interface model. We also assume that temperature drops do not occur across the

thermally conductive lithium electrodes, such that the only temperature drops in the system are across the electrolyte. This assumption is likely valid based on the order of magnitude greater thermal conductivity of lithium of 84.8 W m$^{-1}$ K$^{-1}$,[1] in comparison to the solid electrolytes used, with LPSCl having $k_{\mathrm{LPSCl}} \sim$ 0.5-0.65 W m$^{-1}$ K$^{-1}$,[2,3] and LLZTO having $k_{\mathrm{LLZTO}} \sim$ 1.2-1.4 W-m$^{-1}$ K$^{-1}$.[2,4]

We also define the lithium-ion redox reactions occurring at the lithium electrode-solid electrolyte interfaces. The hot side is defined to be the lithium-ion reduction reaction: $\mathrm{Li}^{+}_{\mathrm{S,H}} + \mathrm{e}^{-}_{\mathrm{E,H}} \rightleftharpoons \mathrm{Li}_{\mathrm{E,H}}$. The cold side is defined to be the lithium-ion oxidation reaction: $\mathrm{Li}_{\mathrm{E,C}} \rightleftharpoons \mathrm{Li}^{+}_{\mathrm{S,C}} + \mathrm{e}^{-}_{\mathrm{E,C}}$. Note that the definition of which side is reduction or oxidation has no impact on the analysis.

When the cell reaches a steady-state during each temperature gradient condition applied at open-circuit conditions, the reaction affinities of the lithium-ion redox reactions are 0 (i.e., the electrochemical potentials of the reactants equals the electrochemical potentials of the products):

$$A_{\mathrm{H}} = \mu^{\mathrm{S}}_{\mathrm{Li^+,H}} + \mu^{\mathrm{E}}_{\mathrm{e^-,H}} - \mu^{\mathrm{E}}_{\mathrm{Li,H}} = 0 \tag{S.1}$$

$$A_{\mathrm{C}} = \mu^{\mathrm{E}}_{\mathrm{Li,C}} - \mu^{\mathrm{S}}_{\mathrm{Li^+,C}} - \mu^{\mathrm{E}}_{\mathrm{e^-,C}} = 0 \tag{S.2}$$

Furthermore, the electrochemical potentials of electrons in the electrode and terminals are equal: $\mu^{\mathrm{E}}_{\mathrm{e^-,H}} = \mu^{\mathrm{T}}_{\mathrm{e^-,H}}$ and $\mu^{\mathrm{E}}_{\mathrm{e^-,C}} = \mu^{\mathrm{T}}_{\mathrm{e^-,C}}$ so that we can replace the electron electrochemical potentials in the lithium metal electrode with the electron electrochemical potentials in the terminals in eq. (S.1) and eq. (S.2). We then fully expand each of the electron electrochemical potential terms, where $\mu_{\mathrm{e^-}} = \mu^{\mathrm{o}}_{\mathrm{e^-}} + \frac{RT}{F} ln\left(\frac{1}{a_{\mathrm{e^-}}}\right) - F\phi$:

$$A_H = \mu^{\mathrm{S}}_{\mathrm{Li^+,H}} + \mu^{\mathrm{o}}_{\mathrm{e^-}} + \frac{RT_{\mathrm{H}}}{F} ln\left(\frac{1}{a_{\mathrm{e^-}}}\right) - F\phi_{\mathrm{e^-_H}^T} - \mu^{\mathrm{E}}_{\mathrm{Li,H}} = 0 \tag{S.3}$$

$$A_C = -\mu^{\mathrm{S}}_{\mathrm{Li^+,C}} - \mu^{\mathrm{o}}_{\mathrm{e^-}} - \frac{RT_{\mathrm{C}}}{F} ln\left(\frac{1}{a_{\mathrm{e^-}}}\right) + F\phi_{\mathrm{e^-_C}^T} + \mu^{\mathrm{E}}_{\mathrm{Li,C}} = 0 \tag{S.4}$$

where $\phi$ is the electric potential. Adding eq. (S.3) and eq. (S.4) together (with $a_{e^-} = 1$), we arrive at:

$$0 = \left(\mu^{S}_{Li^+,H} - \mu^{S}_{Li^+,C}\right) + \left(\mu^{E}_{Li,C} - \mu^{E}_{Li,H}\right) - F\left(\phi_{e^{-T}_{H}} - \phi_{e^{-T}_{C}}\right) \tag{S.5}$$

We then subdivide the remaining lithium-ion and lithium-atom electrochemical potential terms into their electrical (zF$\phi$) and chemical-only (not including electric) potential ($\mu^c$) terms, noting that the c subscript indicates "chemical" rather than "cold":

$$\begin{aligned} 0 = &\left(\mu^{c,S}_{Li^+,H} + z_{Li^+}F\phi_{Li^+,H} - \mu^{c,S}_{Li^+,C} - z_{Li^+}F\phi_{Li^+,C}\right) \\ &+ \left(\mu^{c,E}_{Li,C} + z_{Li}F\phi_{Li,C} - \mu^{c,E}_{Li,H} - z_{Li}F\phi_{Li,H}\right) - F\left(\phi_{e^{-T}_{H}} - \phi_{e^{-T}_{C}}\right) \end{aligned} \tag{S.6}$$

where $z$ is the charge of the respective chemical species (so $z_{Li} = 0$) and $\phi_{Li^+,H}$ is the electric potential of the lithium-ions at the hot-side electrode-electrolyte interface. Simplifying and rearranging:

$$F\left(\phi_{e^{-T}_{C}} - \phi_{e^{-T}_{H}}\right) = F\left(\phi_{Li^+,H} - \phi_{Li^+,C}\right) + \left(\mu^{c,S}_{Li^+,H} - \mu^{c,S}_{Li^+,C}\right) + \left(\mu^{c,E}_{Li,C} - \mu^{c,E}_{Li,H}\right) \tag{S.7}$$

The left-hand side of the equation eq. (S.7) is the measurable open-circuit voltage/energy across the cell and will be represented by $\phi$, the cell voltage. The first grouped term on the right-hand side is the voltage/energy drop across the electrolyte. As we are taking open-circuit voltage measurements, the first-grouped term representing a voltage drop across the cell is regarded as negligible and can be removed from the equation. The next step is to simplify the second and third grouped terms representing the chemical potentials of lithium ions and lithium atoms, respectively. To do so, we perform a taylor expansion around $T_C$ for both grouped terms:

$$\mu^{c,S}_{Li^+,H} \approx \mu^{c,S}_{Li^+,C} + \Delta T * \left(\frac{\delta\mu^{c}_{Li^+}}{\delta T}\right)\Bigg|_{T=T_C} \tag{S.8}$$

$$\mu^{\mathrm{c,S}}_{\mathrm{Li,H}} \approx \mu^{\mathrm{c,S}}_{\mathrm{Li,C}} + \Delta T * \left(\frac{\delta \mu^{\mathrm{c}}_{\mathrm{Li}}}{\delta T}\right)\bigg|_{\mathrm{T=T_C}} \tag{S.9}$$

where $\Delta T = T_{\mathrm{H}} - T_{\mathrm{C}}$. Therefore, eq. (S.7) after removing the first grouped term and substituting eq. (S.8) and eq. (S.9) becomes:

$$F\phi = \Delta T * \left(\frac{\delta \mu^{\mathrm{c}}_{\mathrm{Li^+}}}{\delta T}\right)\bigg|_{\mathrm{T=T_C}} - \Delta T \left(\frac{\delta \mu^{\mathrm{c}}_{\mathrm{Li}}}{\delta T}\right)\bigg|_{\mathrm{T=T_C}} \tag{S.10}$$

We then want to manipulate the derivatives of eq. (S.10) into more useful, measurable forms. To do so, we start with the differential form of the Gibbs free energy, $dG = -SdT + VdP + \Sigma_i \mu_i dN_i$, and perform a Maxwell relation:

$$\left(\frac{\delta S}{\delta N_i}\right)_{T,P,N_{i\neq j}} = -\left(\frac{\delta \mu_i}{\delta T}\right)_{P,N_i} \tag{S.11}$$

We simplify $\left(\frac{\delta S}{\delta N_i}\right)_{T,P,N_{i\neq j}}$ to partial molar entropy, $\overline{S_i}$ and substitute partial molar entropy into equation eq. (S.10), noting that $\mu_i$ in eq. (S.11) is chemical potential and can be directly substituted for $\mu^c_i$ in eq. (S.10). After simplification we arrive at:

$$\frac{\Delta \phi}{\Delta T} = \frac{\left(\overline{S_{\mathrm{Li}}} - \overline{S_{\mathrm{Li^+}}}\right)|_{T=T_C}}{F} \tag{S.12}$$

In literature, the form of eq. (2) from the main text is used:

$$\frac{\delta \phi}{\delta T} = \frac{\left(\overline{S_{\mathrm{Li}}} - \overline{S_{\mathrm{Li^+}}}\right)|_{T=T_C}}{F} \tag{2}$$

where $\overline{S_{\mathrm{Li}}}$ is the partial molar entropy of lithium-atom addition to the lithium-metal electrode, $\overline{S_{\mathrm{Li^+}}}$ is the partial molar entropy of lithium-ion addition to the electrolyte. We further assume that entropy is constant over the temperature range tested, which is shown in figure S1.

An important component of deriving eq. (2) is the assumption of local equilibrium so

that we are able to define thermodynamics properties and make equilibrium thermodynamic conclusions in local subsystems of the inherently nonequilibrium system. For example, we can define local thermodynamic properties such as temperature and chemical potentials, and equate reactant and product chemical potentials at the electrode/electrolyte interfaces, in the nonequilibrium system. This assumption can be made because within typical systems, microscopic scale relaxation times are much faster than macroscopic scale relaxation times.[5]

In addition, the measurements to be used in eq. (2) were taken only at steady-state. In general, the timescales for the temperature gradient and voltage measurements to reach a steady value in LPSCl cells $< 25$ minutes. However, each temperature gradient step was 90 minutes, providing $> 60$ minutes at an observed steady-state. The timescales for the temperature gradient to reach a steady value in LLZTO cells was around 60 minutes. The high signal-to-noise ratios in the LLZTO voltage data made it difficult to determine, but the values were generally equilibrated by 90 minutes into each step. Therefore, as each temperature gradient step was 150 minutes, $> 90$ minutes were spent at a constant temperature gradient, and at least 60 minutes were spent at a relatively constant voltage reading. Given that the cell was at open-circuit conditions and thus there was no current flowing through the cell, in addition to the fact that temperature and voltage conditions were read to be constant, we can infer that the local lithium-ion concentration and temperature profiles were also constant.

## S.2 Derivation of the Composite Solid-Liquid Electrolyte Partial Molar Entropy - Temperature Coefficient Relation

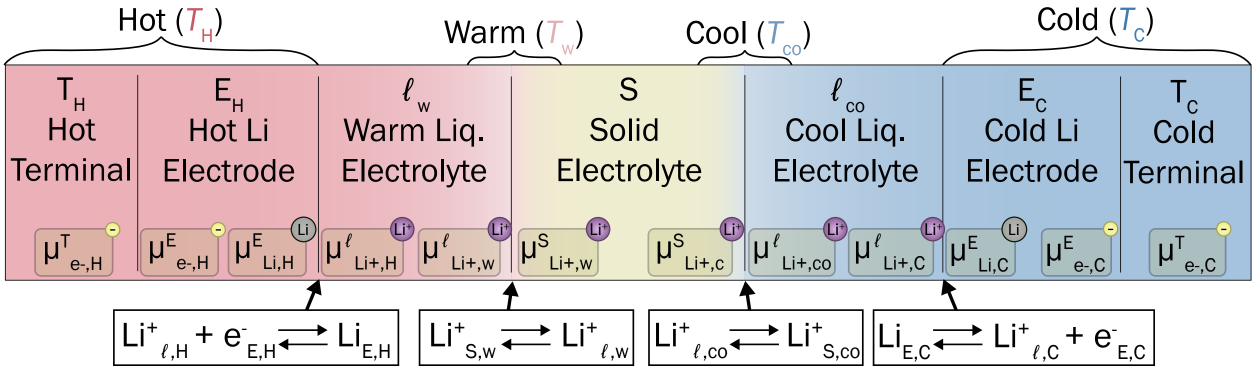


Figure S.2.1: Composite Electrolyte Cell Interface Diagram

The derivation for the composite system closely follows the derivation process performed in S.1. As was done previously, the lithium-ion, lithium-atom, and electron species electrochemical potentials at their respective temperatures are mapped out in the composite electrolyte cell. In this case, there are additional lithium-ion electrochemical potentials for the lithium-ions in liquid electrolyte and additional warm and cool temperatures, $T_w$ and $T_{co}$. The additional electrochemical potentials are defined in Table II.

Table II: Additional Electrochemical Potential Definitions for the Composite Electrolyte Case

| Symbol | Definition |
|---|---|
| $\mu^{\ell}_{Li^+,H}$ | $\mu$ of lithium-ions at the liquid electrolyte's $T_H$ interface |
| $\mu^{\ell}_{Li^+,C}$ | $\mu$ of lithium-ions at the liquid electrolyte's $T_C$ interface |
| $\mu^{\ell}_{Li^+,w}$ | $\mu$ of lithium-ions at the liquid electrolyte's $T_w$ interface |
| $\mu^{\ell}_{Li^+,co}$ | $\mu$ of lithium-ions at the liquid electrolyte's $T_{co}$ interface |
| $\mu^{S}_{Li^+,w}$ | $\mu$ of lithium-ions at the solid electrolyte's $T_w$ interface |
| $\mu^{S}_{Li^+,co}$ | $\mu$ of lithium-ions at the solid electrolyte's $T_{co}$ interface |

The interface model for the composite system shows 4 interfaces, temperatures, and electrochemical reactions that must be considered (Fig. S.2.1). We also again assume that temperature drops occur only in the electrolytes, and not within the lithium-metal electrodes.

At the electrode-liquid electrolyte interface, the same lithium-ion redox reactions occur: $\mathrm{Li}^+_{\ell,\mathrm{H}} + \mathrm{e}^-_{\mathrm{E,H}} \rightleftharpoons \mathrm{Li}_{\mathrm{E,H}}$ and $\mathrm{Li}_{\mathrm{E,C}} \rightleftharpoons \mathrm{Li}^+_{\ell,\mathrm{C}} + \mathrm{e}^-_{\mathrm{E,C}}$. However, there are now also lithium-ion solvation/desolvation "exchange" reactions at the solid-liquid electrolyte interfaces: $\mathrm{Li}^+_{\mathrm{S,w}} \rightleftharpoons \mathrm{Li}^+_{\ell,\mathrm{w}}$ and $\mathrm{Li}^+_{\ell,\mathrm{co}} \rightleftharpoons \mathrm{Li}^+_{\mathrm{S,co}}$. Similar to section S.1, at steady state (and with the local equilibrium assumption), we equate all 4 reaction affinities to 0:

$$A_{\mathrm{H}} = \mu^{\ell}_{\mathrm{Li^+,H}} + \mu^{\mathrm{E}}_{\mathrm{e^-,H}} - \mu^{\mathrm{E}}_{\mathrm{Li,H}} = 0 \tag{S.13}$$

$$A_{\mathrm{C}} = \mu^{\mathrm{E}}_{\mathrm{Li,C}} - \mu^{\ell}_{\mathrm{Li^+,C}} - \mu^{\mathrm{E}}_{\mathrm{e^-,C}} = 0 \tag{S.14}$$

$$A_{\mathrm{w}} = \mu^{\mathrm{S}}_{\mathrm{Li^+,w}} - \mu^{\ell}_{\mathrm{Li^+,w}} = 0 \tag{S.15}$$

$$A_{\mathrm{co}} = \mu^{\ell}_{\mathrm{Li^+,co}} - \mu^{\ell}_{\mathrm{S^+,co}} = 0 \tag{S.16}$$

Following the proecdure outlined in section S.1, we then add equation eq. (S.13)-(S.16) and expand the electron electrochemical potentials of equations:

$$F\left(\phi_{\mathrm{e^-,H}} - \phi_{\mathrm{e^-,C}}\right) = \left(\mu^{\ell}_{\mathrm{Li^+,H}} - \mu^{\ell}_{\mathrm{Li^+,C}}\right) + \left(\mu^{\mathrm{E}}_{\mathrm{Li,C}} - \mu^{\mathrm{E}}_{\mathrm{Li,H}}\right) + \left(\mu^{\mathrm{S}}_{\mathrm{Li^+,w}} - \mu^{\mathrm{S}}_{\mathrm{Li^+,co}}\right) + \left(\mu^{\ell}_{\mathrm{Li^+,co}} - \mu^{\ell}_{\mathrm{Li^+,w}}\right) \tag{S.17}$$

The grouping on the left-hand side of the equation represents the cell voltage and will be represented with $\phi$. We then subdivide the lithium-ion and lithium atom electrochemical potentials to their electrical (zF$\phi$) and chemical-only (not including electric) potential ($\mu^{\mathrm{c}}$) terms:

$$F\phi = \left(\mu^{\mathrm{c},\ell}_{\mathrm{Li^+,H}} - \mu^{\mathrm{c},\ell}_{\mathrm{Li^+,C}}\right) + F\left(\phi^{\ell}_{\mathrm{Li^+,H}} - \phi^{\ell}_{\mathrm{Li^+,C}}\right) + \left(\mu^{\mathrm{c,E}}_{\mathrm{Li,C}} - \mu^{\mathrm{c,E}}_{\mathrm{Li,H}}\right) + \left(\mu^{\mathrm{c,S}}_{\mathrm{Li^+,w}} - \mu^{\mathrm{c,S}}_{\mathrm{Li^+,co}}\right) + F\left(\phi^{\mathrm{S}}_{\mathrm{Li^+,w}} - \phi^{\mathrm{S}}_{\mathrm{Li^+,co}}\right) + \left(\mu^{\mathrm{c},\ell}_{\mathrm{Li^+,co}} - \mu^{\mathrm{c},\ell}_{\mathrm{Li^+,w}}\right) + F\left(\phi^{\ell}_{\mathrm{Li^+,co}} - \phi^{\ell}_{\mathrm{Li^+,w}}\right) \tag{S.18}$$

The second, fifth, and seventh terms on the right hand side represent the potential drop

across the various electrolyte layers, and can be treated as negligible in our open-circuit voltage measurements. We then rearrange the liquid-electrolyte lithium-ion electrochemical potentials to that hot and warm temperatures are together and cold and cool temperatures are together:

$$F\phi = \left(\mu^{\mathrm{c,E}}_{\mathrm{Li,C}} - \mu^{\mathrm{c,E}}_{\mathrm{Li,H}}\right) + \left(\mu^{\mathrm{c},\ell}_{\mathrm{Li^+,H}} - \mu^{\mathrm{c},\ell}_{\mathrm{Li^+,w}}\right) + \left(\mu^{\mathrm{c,S}}_{\mathrm{Li^+,w}} - \mu^{\mathrm{c,S}}_{\mathrm{Li^+,co}}\right) + \left(\mu^{\mathrm{c},\ell}_{\mathrm{Li^+,co}} - \mu^{\mathrm{c},\ell}_{\mathrm{Li^+,C}}\right) \quad (S.19)$$

We then simplify all terms on the right-hand side of eq. (S.19) using Taylor Expansions:

$$\mu^{\mathrm{c,E}}_{\mathrm{Li,H}} \approx \mu^{\mathrm{c,E}}_{\mathrm{Li,C}} + \Delta T_{\text{H-C}} * \left(\frac{\delta \mu^{\mathrm{c}}_{\mathrm{Li}}}{\delta T}\right)\bigg|_{\mathrm{T=T_C}} \quad (S.20)$$

$$\mu^{\mathrm{c},\ell}_{\mathrm{Li^+,H}} \approx \mu^{\mathrm{c},\ell}_{\mathrm{Li^+,w}} + \Delta T_{\text{H-w}} * \left(\frac{\delta \mu^{\mathrm{c},\ell}_{\mathrm{Li^+}}}{\delta T}\right)\bigg|_{\mathrm{T=T_w}} \quad (S.21)$$

$$\mu^{\mathrm{c,S}}_{\mathrm{Li^+,w}} \approx \mu^{\mathrm{c,S}}_{\mathrm{Li^+,co}} + \Delta T_{\text{w-co}} * \left(\frac{\delta \mu^{\mathrm{c},\ell}_{\mathrm{Li^+}}}{\delta T}\right)\bigg|_{\mathrm{T=T_{co}}} \quad (S.22)$$

$$\mu^{\mathrm{c},\ell}_{\mathrm{Li^+,co}} \approx \mu^{\mathrm{c},\ell}_{\mathrm{Li^+,C}} + \Delta T_{\text{co-C}} * \left(\frac{\delta \mu^{\mathrm{c},\ell}_{\mathrm{Li^+}}}{\delta T}\right)\bigg|_{\mathrm{T=T_C}} \quad (S.23)$$

where $\Delta T_{H-C} = T_{\mathrm{H}} - T_{\mathrm{C}}$, $\Delta T_{H-w} = T_{\mathrm{H}} - T_{\mathrm{w}}$, $\Delta T_{w-co} = T_{\mathrm{w}} - T_{\mathrm{co}}$, and $\Delta T_{co-C} = T_{\mathrm{co}} - T_{\mathrm{C}}$. Although eq. (S.21) and eq. (S.22) reference the derivative to a temperature other than $T_{\mathrm{C}}$, the same constant entropy assumption keeps the derivation accurate. In fact, the temperature difference between hot and warm in eq. (S.21) and between warm and cool in eq. (S.22) is less than the temperature difference between the hot and cold, meaning the constant entropy assumption actually needs to hold over a smaller temperature difference.

Using the relationship from eq. (S.11), we can then simplify eq. (S.19) to eq. (3) from the main text by relating the temperature drops across each electrolyte to the cell voltage change with partial molar entropies as parameters:

$$\phi = \frac{1}{F}[\Delta T_{\text{H-C}}\overline{S_{\text{Li}}} - \Delta T_{\text{w-co}}\overline{S^{\text{S}}_{\text{Li}^+}} - (\Delta T_{\text{H-w}} + \Delta T_{\text{co-C}})\overline{S^{\ell}_{\text{Li}^+}}] \tag{3}$$

where $\overline{S_{\text{Li}}}$ is the partial molar entropy of lithium-atom addition to the lithium-metal electrode, $\overline{S^{S}_{\text{Li}^+}}$ is the partial molar entropy of lithium-ion addition to the wetted solid electrolyte, and $\overline{S^{\ell}_{\text{Li}^+}}$ is the partial molar entropy of lithium-ion addition to the liquid electrolyte.

Again, the assumption of local equilibrium is crucial. The same reasoning as used in Section S.1 is applied to making the local equilibrium assumption in this derivation.

## S.3 Incorporation of 1-D Heat Transfer into the Composite System Temperature Coefficient

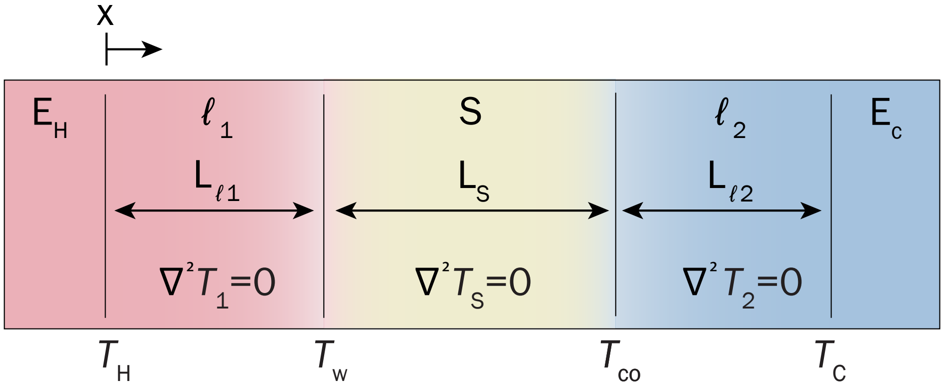


Figure S.3.1: 1-D Heat Conduction Model

To incorporate system thickness (configuration) and thermal conductivity properties into eq. (3) and eliminate the need to measure the internal temperatures $T_{\mathrm{w}}$ and $T_{\mathrm{co}}$, the cell voltage-partial molar entropy equation, a 1-D heat conduction problem is set up (Fig. S.3.1). The focus is placed on the three electrolyte components where temperature drops occur across their thickness, $L_i$. $\ell_1$ refers to the hot/warm liquid electrolyte layer, S refers to the warm/cool solid electrolyte layer, and $\ell_2$ refers to the cool/cold electrolyte layer. $k_i$ are the thermal conductivites for each layer. For a steady-state, static system, the heat transport equation is $\nabla^2 T = 0$. This equation is applied to each electrolyte component of the cell, giving a system of three second-order differential equations:

$$1) \quad \nabla^2 T_1 = 0$$

$$2) \quad \nabla^2 T_{\mathrm{S}} = 0$$

$$3) \quad \nabla^2 T_2 = 0$$

To solve the system of three second-order differential equations, six boundary conditions

are defined:

$$1) \quad T_{\ell_1}|_{x=0} = T_{\mathrm{H}}$$

$$2) \quad T_{\ell_2}|_{\mathrm{x=L_{\ell_1}+L_{\mathit{S}}+L_{\ell_2}}} = T_{\mathrm{C}}$$

$$3) \quad T_{\ell_1}|_{\mathrm{x=L_{\ell_1}}} = T_{\mathrm{S}}|_{\mathrm{x=L_{\ell_1}}}$$

$$4) \quad T_{\mathrm{S}}|_{\mathrm{x=L_{\ell_1}+L_{\mathit{S}}}} = T_{\ell_2}|_{\mathrm{x=L_{\ell_1}+L_{\mathit{S}}}}$$

$$5) \quad -k_{\ell_1}\left.\frac{dT_{\ell_1}}{dx}\right|_{\mathrm{x=L_{\ell_1}}} = -k_S\left.\frac{dT_{\mathrm{S}}}{dx}\right|_{\mathrm{x=L_{\ell_1}}}$$

$$6) \quad -k_S\left.\frac{dT_{\mathrm{S}}}{dx}\right|_{\mathrm{x=L_{\ell_1}+L_{\mathit{S}}}} = -k_{\ell_2}\left.\frac{dT_{\ell_2}}{dx}\right|_{\mathrm{x=L_{\ell_1}+L_{\mathit{S}}}}$$

Boundary conditions 1-4 encapsulate the equal temperatures at the 4 interfaces and conditions 5-6 come from symmetric heat transfer (defined with Fourier's Law of Heat Conduction) across the middle interfaces. Then, the system of second order differential equations can be solved to find $T_w$ and $T_c$:

$$T_{\ell_1}|_{\mathrm{x=L_{\ell_1}}} = T_{\mathrm{w}} = T_{\mathrm{H}} - \frac{k_{\mathrm{S}} L_{\ell_1}\left(T_{\mathrm{H}} - T_{\mathrm{C}}\right)}{k_{\mathrm{S}}\left(\mathrm{L}_{\ell_1} + \mathrm{L}_{\ell_2}\right) + k_\ell L_{\mathrm{S}}} \tag{S.24}$$

$$T_{\ell_1}|_{\mathrm{x=L_{\ell_1}+L_S}} = T_{\mathrm{co}} = \frac{k_{\mathrm{S}} L_{\ell_1} T_{\mathrm{C}} + k_\ell L_{\mathrm{S}} T_{\mathrm{C}} + k_{\mathrm{S}} L_{\ell_2} T_{\mathrm{H}}}{k_{\mathrm{S}}\left(\mathrm{L}_{\ell_1} + \mathrm{L}_{\ell_2}\right) + k_\ell L_{\mathrm{S}}} \tag{S.25}$$

We then substitute eq. (S.24) and eq. (S.25) into eq. (3) via $\Delta T_{\text{H-w}}$, $\Delta T_{\text{w-co}}$, and $\Delta T_{\text{co-C}}$. After simplification, we arrive at eq. (S.26):

$$\phi = \frac{1}{F}\left[\frac{\left(L_{\mathrm{S}} k_\ell + \left(L_{\ell_{\mathrm{w}}} + L_{\ell_{\mathrm{c}}}\right) k_{\mathrm{S}}\right)\overline{S_{\mathrm{Li}}} - L_{\mathrm{S}} k_\ell \overline{S^{\mathrm{S}}_{\mathrm{Li^+}}} - \left(L_{\ell_{\mathrm{w}}} + L_{\ell_{\mathrm{c}}}\right) k_{\mathrm{S}} \overline{S^{\ell}_{\mathrm{Li^+}}}}{L_{\mathrm{S}} k_\ell + \left(L_{\ell_{\mathrm{w}}} + L_{\ell_{\mathrm{c}}}\right) k_{\mathrm{S}}}\right]\left(T_{\mathrm{H}} - T_{\mathrm{C}}\right) \tag{S.26}$$

## S.4 Non-Dimensionalization of the Composite System Temperature Coefficient Model

We then non-dimensionalize eq. (S.26). We define our non-dimensional values as such:

$$\text{Temperature Difference:}\quad \theta = \frac{T_H - T_C}{T_C} = \frac{\Delta T}{T_C}$$

$$\text{Solid-Electrolyte Length:}\quad \mathcal{L}_\mathrm{S} = \frac{L_\mathrm{S}}{L_\mathrm{c}}\quad \text{, where } L_\mathrm{c} \text{ is a characteristic cell length}$$

$$\text{Liquid-Electrolyte Lengths:}\quad \mathcal{L}_{\ell_\mathrm{w}} = \frac{L_{\ell_\mathrm{w}}}{L_\mathrm{c}} \quad \text{and} \quad \mathcal{L}_{\ell_\mathrm{c}} = \frac{L_{\ell_\mathrm{c}}}{L_\mathrm{c}}$$

$$\text{Thermal conductivity:}\quad \kappa = \frac{k_\ell}{k_\mathrm{S}}$$

$$\text{Lithium Atom Partial Molar Entropy:}\quad \overline{S^o_\mathrm{Li}} = \frac{\overline{S_\mathrm{Li}}}{R}$$

$$\text{Lithium-ion Partial Molar Entropy in the Liquid Electrolyte:}\quad \overline{S^{\mathrm{o},\ell}_{\mathrm{Li}^+}} = \frac{\overline{S^{\ell}_{\mathrm{Li}^+}}}{\overline{S_\mathrm{Li}}}$$

$$\text{Lithium-ion Partial Molar Entropy in the Solid Electrolyte:}\quad \overline{S^{\mathrm{o,S}}_{\mathrm{Li}^+}} = \frac{\overline{S^{\mathrm{S}}_{\mathrm{Li}^+}}}{\overline{S_\mathrm{Li}}}$$

These non-dimensional values appear in eq. (S.26) after factoring out $S_\mathrm{Li}$, $k_\mathrm{S}$, and $L$ from the numerator of the bracket, while also dividing both sides by the gas constant $R$ and $T_\mathrm{C}$. The non-dimensionalized form thus becomes

$$\frac{F}{RT_\mathrm{C}}\phi = \overline{S^o_\mathrm{Li}}\left[\frac{(\mathcal{L}_{\ell_\mathrm{w}} + \mathcal{L}_{\ell_\mathrm{c}})\left(1 - \overline{S^{\mathrm{o},\ell}_{\mathrm{Li}^+}}\right) + \kappa\mathcal{L}_\mathrm{S}\left(1 - \overline{S^{o,\mathrm{S}}_{\mathrm{Li}^+}}\right)}{\mathcal{L}_{\ell_\mathrm{w}} + \mathcal{L}_{\ell_\mathrm{c}} + \kappa\mathcal{L}_\mathrm{S}}\right]\theta \tag{S.27}$$

For the final part of the study, we are interested in varying the solid electrolyte length, so we want the non-dimensional lengths in eq. (S.27) to only be in terms of $\mathcal{L}_\mathrm{S}$. We note that the non-dimensional electrolyte lengths are linked via the constraint $\frac{\mathcal{L}_{\ell_1}+\mathcal{L}_\mathrm{S}+\mathcal{L}_{\ell_1}}{\mathcal{L}_\mathrm{c}} = 1$. Thus, we know that $\mathcal{L}_{\ell_1} + \mathcal{L}_{\ell_2} = 1 - \mathcal{L}_\mathrm{S}$. With the assumption that $\mathcal{L}_{\ell_1} = \mathcal{L}_{\ell_2}$, which should be the case based on the construction of the composite cell (equal number of glass fiber pieces

on each side, soaked with the same amount of electrolyte, and compressed under the same pressure), we can substitute that relation into eq. (S.27), divide both sides by $\theta$ and get

$$\frac{F}{RT_{\mathrm{C}}}\frac{\phi}{\theta} = \overline{S^{\mathrm{o}}_{\mathrm{Li}}}\left[\frac{(1-\mathcal{L}_{\mathrm{S}})\left(1-\overline{S^{\mathrm{o},\ell}_{\mathrm{Li}^+}}\right)+\kappa\mathcal{L}_{\mathrm{S}}\left(1-\overline{S^{\mathrm{o,S}}_{Li^+}}\right)}{1-\mathcal{L}_{\mathrm{S}}+\kappa\mathcal{L}_{\mathrm{S}}}\right] \tag{4}$$

The left hand side is the "non-dimensional temperature coefficient".

Table III: Values used in Fig. 4A

| **Cell Parameter** | **Value** |
|---|---|
| $L_{\mathrm{S}}$ | Varies: 0.90, 1.15, 1.30, 1.50, 1.67, 1.89 mm |
| $L_\ell$ | 0.28 mm (fitted measured temperatures to equations S.24, S.25) |
| $\overline{S_{\mathrm{Li}}}$ | 29.1 J mol$^{-1}$ K$^{-1}$ |
| $\overline{S^{\mathrm{o}}_{\mathrm{Li}}}$ | 3.5 |
| $\overline{S^{\mathrm{S}}_{\mathrm{Li}^+}}$ | $-26.99$ J mol$^{-1}$ K$^{-1}$ (Fig. S6) |
| $\overline{S^{\mathrm{o,S}}_{\mathrm{Li}^+}}$ | $-0.927$ |
| $\overline{S^{\ell}_{\mathrm{Li}^+}}$ | $-76.39$ J mol$^{-1}$ K$^{-1}$ (Fig. S6) |
| $\overline{S^{\mathrm{o},\ell}_{\mathrm{Li}^+}}$ | $-2.625$ |
| $k_{\mathrm{S}}$ | 0.5 W m$^{-1}$ K$^{-1}$ ref.[2,3] |
| $k_\ell$ | 0.2 W m$^{-1}$ K$^{-1}$ ref.[6,7] |
| $\kappa$ | 0.4 |

# References


(1) Dean, J. *Lange's Handbook of Chemistry*, 15th ed.; McGraw-Hill, 1998.

(2) Cheng, Z.; Zahiri, B.; Ji, X.; Chen, C.; Chalise, D.; Braun, P. V.; Cahill, D. G. Good Solid-State Electrolytes Have Low, Glass-Like Thermal Conductivity. *Small* **2021**, *17*.

(3) Böger, T.; Bernges, T.; Li, Y.; Canepa, P.; Zeier, W. G. Thermal Conductivities of Lithium-Ion-Conducting Solid Electrolytes. *ACS Applied Energy Materials* **2023**, *6*, 10704–10712.

(4) Wang, Y.; Li, S.; Wu, N.; Jia, Q.; Hoke, T.; Shi, L.; Li, Y.; Chen, X. Thermal properties and lattice anharmonicity of Li-ion conducting garnet solid electrolyte Li6.5La3Zr1.5Ta0.5O12. *Journal of Materials Chemistry A* **2024**, *12*, 18248–18257.

(5) Vilar, J. M.; Rubi, J. Thermodynamics "beyond" local equilibrium. *Proceedings of the National Academy of Sciences* **2001**, *98*, 11081–11084.

(6) Yaws, C. *Handbook of Thermal Conductivity*; Gulf Professional Publishing, 1995; Vol. 3.

(7) Munteshari, O.; Lau, J.; Krishnan, A.; Dunn, B.; Pilon, L. Isothermal calorimeter for measurements of time-dependent heat generation rate in individual supercapacitor electrodes. *Journal of Power Sources* **2018**, *374*, 257–268.
